# Tailoring Morphology and Wetting Behavior of Films of Ionic Liquid Mixtures

**DOI:** 10.1021/acs.langmuir.5c00653

**Published:** 2025-03-27

**Authors:** Soraia R. M. R. Silva, Rita M. Carvalho, Oleksandr Bondarchuk, Gonçalo N. P. Oliveira, João P. Araújo, Margarida Bastos, Luís M. N. B. F. Santos, José C. S. Costa

**Affiliations:** † CIQUP/Institute of Molecular Sciences (IMS), Departamento de Química e Bioquímica, Faculdade de Ciências, Universidade do Porto, Rua do Campo Alegre, s/n, 4169-007 Porto, Portugal; ‡ 246702 International Iberian Nanotechnology Laboratory, Av. Mestre José Veiga, s/n, 4715-330 Braga, Portugal; § SPIN-Lab Centre for Microscopic Research on Matter, University of Silesia in Katowice, 75 Pułku Piechoty Str. 1A, Chorzów 41-500, Poland; ∥ Institute of Chemistry, University of Silesia in Katowice, 9 Szkolna Str., Katowice 40-006, Poland; ⊥ IFIMUP, Instituto de Física de Materiais Avançados, Nanotecnologia e Fotónica, Departamento de Física e Astronomia, Faculdade de Ciências, Universidade do Porto, Rua do Campo Alegre, s/n, 4169-007 Porto, Portugal

## Abstract

Extensive research has focused on films formed by pure ionic liquids (ILs). However, growing interest in IL mixtures and their synergistic properties presents new opportunities for targeted applications and fundamental scientific investigations. This study explores the morphology of films composed of mixtures of two ILs, [C_2_C_1_im]­[OTf] and [C_8_C_1_im]­[OTf], co-deposited via physical vapor deposition (PVD)/vacuum thermal evaporation. The primary objective was understanding how varying the IL ratio influences droplet formation, surface coverage, and overall film structure. Thin-film growth was examined on glass substrates coated with indium tin oxide (ITO) and ITO/glass surfaces coated with metallic films (Au and Ag). Film morphology was characterized using optical and high-resolution scanning electron microscopy (SEM), while elemental composition was analyzed via X-ray photoelectron spectroscopy (XPS). The results show that IL mixture morphology is strongly influenced by both IL composition and substrate type. Increasing [C_8_C_1_im]­[OTf] content led to larger microstructures due to improved wetting, particularly on Au surfaces, resulting in nearly fully coalesced films. Metallic surfaces near ITO significantly impacted droplet behavior, with ILs exhibiting a strong affinity for metals, especially when the long-chain IL dominated the mixture. The IL-assisted crystallization of rubrene, a high-performance organic semiconductor (OSC) that typically exhibits poor crystallinity when deposited via PVD, highlights the potential of IL mixtures to enhance organic film quality. X-ray diffraction (XRD) confirmed that [C_2_C_1_im]­[OTf] and [C_8_C_1_im]­[OTf] mixtures significantly improved rubrene crystallinity, demonstrating their potential to create an optimal environment for OSC solubility and crystallization.

## Graphical abstract

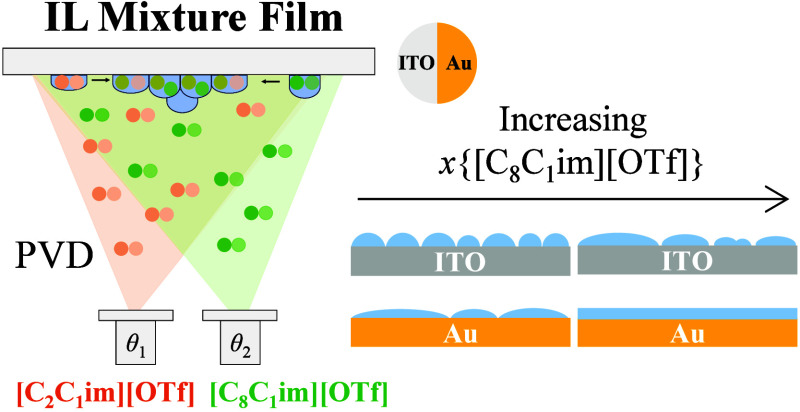

## Introduction

The scientific community has recently shown increasing interest in ionic liquid (IL) films due to their high potential as interfacial materials. ILs have found applications in widely diverse fields, including materials chemistry and physics, chemical engineering, and environmental science.^1–5^ ILs offer structural versatility, excellent solvent capabilities, and environmental advantages, positioning them as valuable tools in various branches of chemistry. Their unique physical and chemical properties—such as high ionic conductivity, exceptional thermal and chemical stability, low vapor pressure at room temperature, a wide electrochemical window, and remarkable wettability—make them invaluable in many areas.^6–11^

ILs form well-organized nanostructures with polar and nonpolar domains significantly influencing their bulk and surface properties.^6,12^ The nature of the cation, anion, and the length of the cation’s alkyl chain can be systematically adjusted to tune characteristics such as wettability and surface energy.^13–17^ The low vapor pressure of ILs makes their use superior to conventional volatile organic compounds as regarding toxicity concerns and enables these materials to be used in vacuum applications.^4,18–22^

Presently, there is a growing interest in IL mixtures, as the composition of IL mixtures can impart distinctive properties and functionalities that differ from those of individual ILs. By combining varying cations and anions, diverse structural organization, molecular motion, and intermolecular interactions can be achieved at a molecular level.^23,24^ This knowledge forms the foundation for tailoring IL mixtures to meet the requirements for various applications, such as gas separation processes (CO_2_ capture) and energy storage devices (such as batteries and supercapacitors).^25–30^

In the realm of thin films/coatings, ILs have captured considerable research interest, so far mainly focusing on pure ILs.^4,13,14,18,22,31–45^ Exploring IL mixture films will provide insight into their behavior and properties, raising fundamental scientific questions that will drive innovative advancements in thin-film technologies. In the field of optoelectronics, thin IL films have been shown to be promising in the development of more efficient organic semiconductors (OSCs).^4,46–48^ Controlling polymorphism and grain growth in OSC thin films is crucial due to its significant impact on OSC conductivity.^49–53^ As regarding these aspects, IL-assisted vapor deposition is a very powerful technique as it uses ILs as solvents or templates during thin film fabrication. With this method, the growth of single, directionally oriented OSC crystals produced via physical vapor deposition (PVD) is significantly enhanced.^4,18,21,22,46–48^

Previous work has extensively investigated nucleation and growth mechanisms of various imidazolium-based ILs deposited via vacuum thermal evaporation technique.^4,13,14,18,22,31–45^ Studies at the nanoscale, particularly on metal surfaces, have highlighted the influence of IL ion pairs adsorption and the structuring of the first monolayer (ML) on IL film properties.^31,37–42^ Several studies have explored how the structuring of ILs influences film morphology by using different cation–anion pairs and varying the alkyl chain sizes associated with the cationic group and their symmetry.^13,14,34^ Additionally, it was found that the chemical nature of the substrate, its roughness, potential adventitious carbon contaminations, and deposition conditions all play crucial roles in IL nucleation, droplet formation, and spreading on solid surfaces.^13,14,36,44^ The formation of IL films is influenced by three main factors: the surface diffusion of ion pairs, the availability of minimum free area for nucleation (MFAN), and coalescence mechanisms.^33,54–56^ Initially, ion pairs seek favorable binding sites on the surface and diffuse until they settle in optimal locations where they adsorb, forming initial clusters or *nuclei*. This stage, known as nucleation, is greatly influenced by the affinity of the IL with the substrate, which defines the MFAN.^33^ As described by Matsumoto et al., the assumption that individual IL pairs are initially isolated before being deposited onto the substrate should be approached with caution. In fact, depending on the deposition conditions, IL pairs may aggregate into cluster-like particles in the vapor phase before being deposited onto the substrate. Therefore, it is possible that a mixed phenomenon occurs, where both isolated IL pairs and aggregated clusters contribute to the deposition process, depending on the specific conditions.^57^ As the clusters grow on the surface, they merge with neighboring clusters, undergo coalescence, and enable subsequent film growth. Strong adhesion to the substrate promotes layer-by-layer (2D) growth, whereas weaker adhesion leads to island-like (3D) growth.^33^ In some cases, ILs exhibit mixed growth on metal surfaces, with an initial 2D wetting layer formation followed by 3D growth. In this particular case, the ion pairs spread into a continuous film before transitioning into droplet formation.^38^ From a thermodynamic perspective, the morphology of the deposited IL droplets not only depends on the surface tension of the IL (γ_l–v_) but also on the surface tension of the substrate (γ_s–v_) and the interfacial substrate-IL tension (γ_s–l_). For a macroscopic system with chemically inert, homogeneous, and rigid substrates, the droplet contact angle, θ_c_, can be calculated using Young’s equation, as shown in eq 1.^58^ For excellent wettability, γ_l–v_ must be lower than {γ_s–v_ – γ_s–l}_.

$$\cos\left(\theta_{c}\right)=\frac{\gamma_{s-v}-\gamma_{s-l}}{\gamma_{l-v}}$$ 1

Many studies focus on imidazolium-based ILs, and it has been observed that ILs with long alkyl chains tend to form a uniform film when deposited on metal surfaces, particularly on gold. In contrast to short-chain ILs, which exhibit a pronounced tendency for island growth, long-chain ILs demonstrate clear 2D layer growth on various surfaces, as observed, for example, with [C_8_C_1_im]­[NTf_2_] and [C_8_C_1_im]­[OTf] deposited on gold.^13,14^ This behavior is attributed to the lower values of γ_l–v_ and γ_s–l_ for the long-chain ILs. Compared to pure ILs, fewer studies have focused on the surface and interfacial properties of IL mixtures. Steinrück et al. reported that codeposited mixed IL films on metal surfaces exhibit complex interfacial behavior.^59,60^

The present work focuses on forming IL mixture films by combining two imidazolium-based ILs: the short-chain [C_2_C_1_im]­[OTf] and the long-chain [C_8_C_1_im]­[OTf]. The molecular structures of these ILs are depicted in Figure 1. This study aims to elucidate the morphological differences found in thin films formed by the co-evaporation of these imidazolium-based ILs in varying proportions. The study was conducted under uniform deposition conditions across diverse substrates, including transparent conductive oxides, silver, and gold. Both the composition of the IL mixture and the substrate type were found to influence the film morphology and surface wettability significantly. Furthermore, the study examined the vacuum deposition process of rubrene on substrates previously coated with IL films. Rubrene, known for its high performance as an OSC but limited crystallization efficiency via vacuum thermal evaporation on solid surfaces,^52,61,62^ serves as an ideal test case to demonstrate the capabilities of IL mixture films as a promising medium for the enhanced crystallization of OSCs.

**Figure 1:**
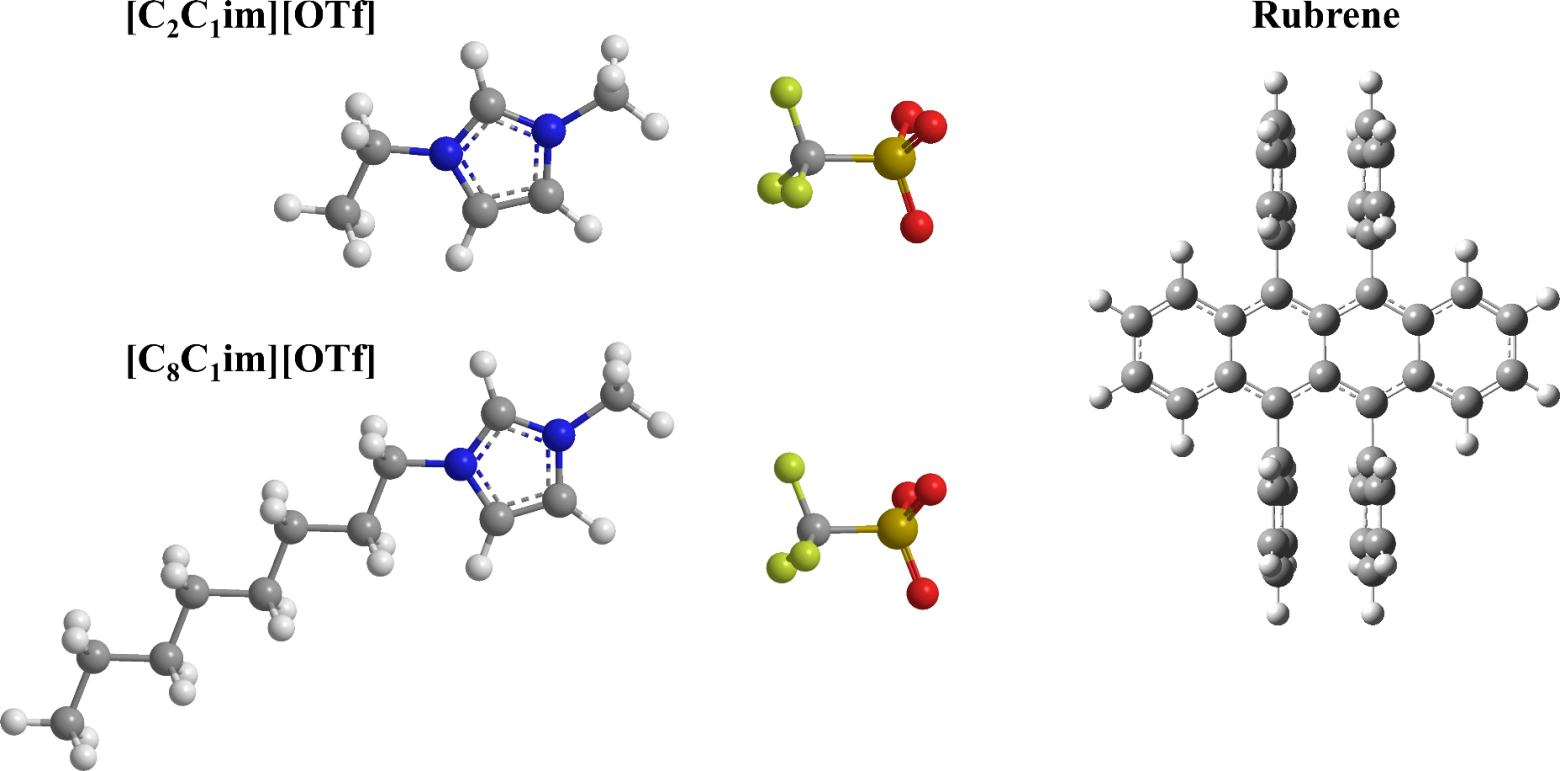
Molecular structures of the studied compounds: 1-ethyl-3-methylimidazolium triflate, [C_2_C_1_im]­[OTf]; 1-methyl-3-octylimidazolium triflate, [C_8_C_1_im]­[OTf]; 5,6,11,12-tetraphenyltetracene (rubrene).

## Experimental Details

### Reagents

1-Methyl-3-octylimidazolium triflate, [C_8_C_1_im]­[OTf] (CAS No. 403842-84-2), and 1-ethyl-3-methylimidazolium triflate, [C_2_C_1_im]­[OTf] (CAS No. 145022-44-2), were commercially obtained from IoLiTec GmbH with a state purity of 99%. Rubrene (CAS No. 517-51-1) was purchased from Sigma-Aldrich with 98% purity. Before initiating the deposition experiments, the ILs were dried to reduce water and other volatile contents to ppm range values. This was achieved under reduced pressure (<10 Pa) with continuous stirring at 373 K for at least 48 h. Additionally, within the vacuum chamber of the deposition system, the ILs were exposed to higher temperatures (423 K) to ensure that any potential nonvolatile impurities, such as polymeric derivatives in the ppm range, would not interfere with the deposited films. Table S1 in the Supporting Information (SI) provides some relevant properties of the ILs studied. Rubrene was washed twice with hot methanol and then purified by successive sublimations under reduced pressure. The purity of the rubrene sample was confirmed by gas chromatography using an HP 4890 apparatus equipped with an HP-5 column (cross-linked, 5% diphenyl, and 95% dimethylpolysiloxane). No impurities were detected, and the purity grade was assigned as >99.9%.

### Substrates

The deposition of IL films (pure samples or mixtures) was performed on indium tin oxide-coated glass (ITO/glass), ITO/glass substrates partially coated with silver (Ag/ITO/glass), and ITO/glass substrates partially coated with gold (Au/ITO/glass). The ITO/glass substrates, with dimensions of 10 mm × 10 mm × 1.1 mm, were commercially obtained from Praezisions Glas & Optik GmbH. The ITO films have a thickness of approximately 180 nm. Before the deposition experiments, the ITO/glass substrates were washed in an ultrasonic bath with high-purity ethanol. Subsequently, the substrates were dried using argon and stored in a desiccator. The ITO substrates underwent a sputtering process to obtain the metal surfaces (Ag/ITO and Au/ITO), coating the ITO surfaces with a metal film of 100 nm thickness. This was achieved using a Cressington 108 Auto Sputter Coater, which employs direct current (DC) magnetron sputtering. The vacuum chamber was filled with pure argon, and an electric field (discharging current of 40 mA) was applied to generate plasma, enabling the sputtering onto the ITO surfaces. High-purity (>99.9%) metal targets were utilized in this study. The thickness of the deposited metal films was monitored using a quartz crystal microbalance (QCM). Each ITO substrate was divided into two halves. Half of each substrate was covered with Kapton tape, while the other half remained exposed. Metal (Au or Ag) was sputtered onto the exposed ITO and the Kapton taped area. After the sputtering process, the Kapton tape was removed. This approach ensured that each substrate had two distinct surfaces: one with metal and the other with only ITO (see Figure 2 for a schematic representation). These substrates were then used for the deposition of ILs, enabling comparative analysis under controlled conditions and providing the additional advantage of examining the interface between the ITO and the metal. To maintain sample integrity, all substrates used in the study were stored in a desiccator before the deposition experiments to minimize exposure to air and potential contamination.

**Figure 2:**
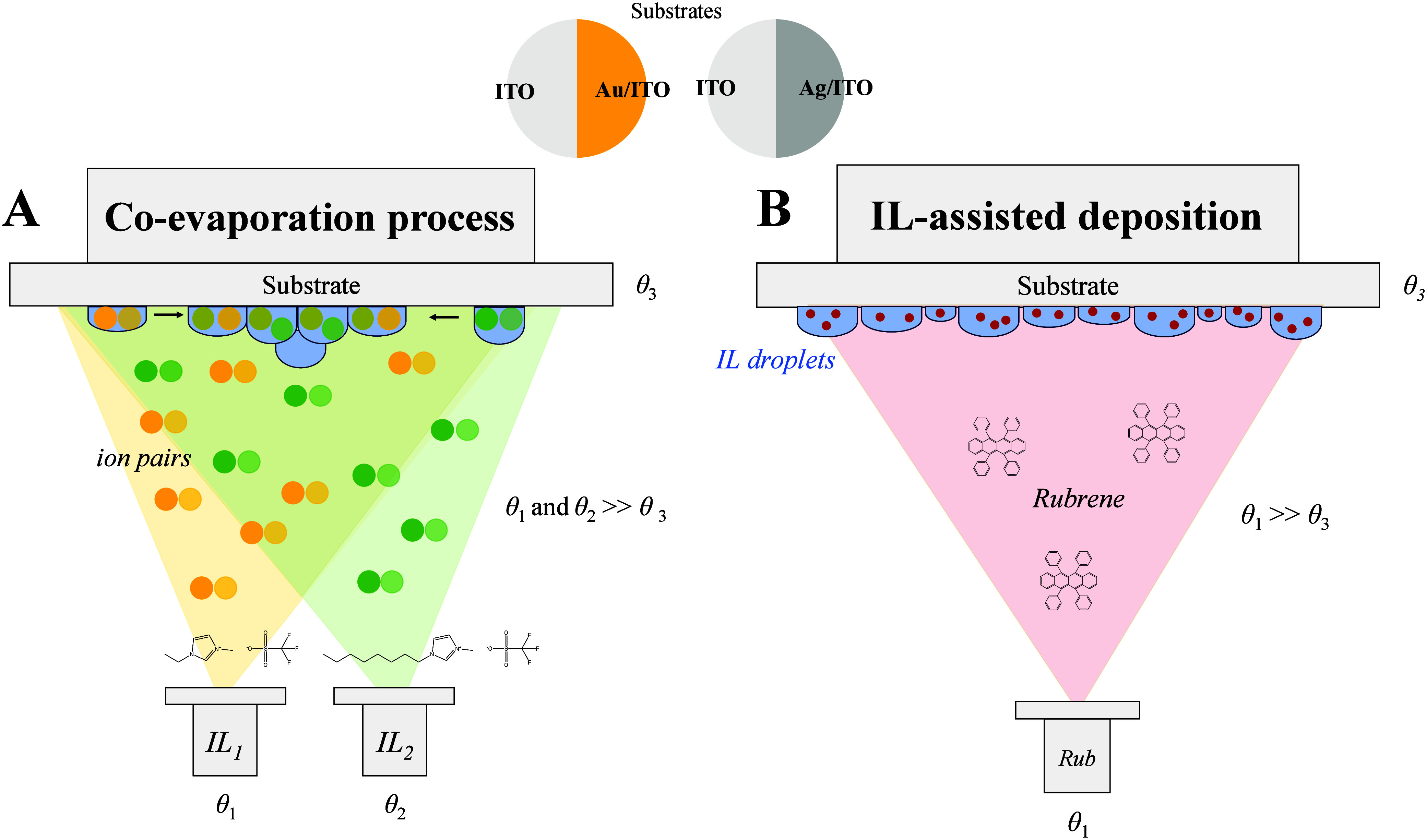
Schematic representation of the co-deposition process of [C_2_C_1_im]­[OTf] and [C_8_C_1_im]­[OTf] (A) and the deposition of Rubrene (Rub) by the process of ionic liquid-assisted vapor deposition (B). θ_1_ and θ_2_ represent the temperatures of the evaporation sources, and θ_3_ represents the substrate temperature.

### Co-evaporation of [C_2_C_1_im]­[OTf] and [C_8_C_1_im]­[OTf]

Thin films of [C_8_C_1_im]­[OTf] and [C_2_C_1_im]­[OTf], as well as mixtures of these ILs, were fabricated using PVD through a vacuum thermal evaporation process with customized equipment developed in our laboratory.^63^ The Knudsen effusion cells allow precise mass flow rate control.^33,63^ The detailed methodology has been extensively described in recent publications.^13,14,18,33–36,44,48^ The deposition experiments were conducted under reduced pressure (*p* < 10^–4^ Pa). The temperature control of the Knudsen cell precisely regulates the gradual vaporization of the IL through the effusion orifice. The deposition system incorporates multiple Knudsen effusion cells with independent temperature control. The formation of a mixture film involving [C_8_C_1_im]­[OTf] and [C_2_C_1_im]­[OTf] was achieved through a simultaneous deposition (co-evaporation) process from two independent evaporation sources (see Figure 2A). We consider this strategy the more practical approach, as it allowed precise control over the mass flow rate of each IL using separate Knudsen effusion cells. Thermal evaporation from a preprepared mixture could potentially alter the energy required for evaporation, depending on the specific interactions between the components of the mixture. Furthermore, the simultaneous deposition method required significantly less IL compared to preparing mixtures with varying mole fractions prior to evaporation. Films with varying proportions of each IL were obtained by setting a global deposition rate and adjusting the temperature of each Knudsen cell containing the IL. The deposition mass flow rate (φ) was determined using a modified form of the Knudsen equation, as expressed in eq 2.

$$\varphi_{s}=g\varphi_{\mathrm{Kc}}=gpw_{o}\sqrt{\frac{M}{2\pi RT}}=g\frac{m}{A_{o}t}$$ 2

The mass flow rates φ_s_ and φ_Kc_ represent the rates at the substrate surface and from the Knudsen cell orifice, respectively. φ_s_ was monitored using a quartz crystal microbalance (QCM, Inficon model STM-2), and φ_Kc_ was determined by the Knudsen equation. φ_Kc_ was crucial for controlling the proportion of each IL in the mixture. The geometric factor (*g*), derived as φ_s_/φ_Kc_, depends on the distance between the Knudsen cell and the substrate. In addition, *p* represents the equilibrium vapor pressure of the IL, *w*_0_ is the transmission probability factor, *M* is the molar mass of the effused IL vapor, *T* is the evaporation temperature, *m* is the mass of the evaporated sample, and *A*_0_ is the area of the Knudsen cell orifice. The details of the vacuum thermal evaporation methodology are presented in Figures S1–S7 of the Supporting Information.

Different amounts of ILs were deposited as either pure IL films or IL mixture films onto ITO, Ag/ITO, and Au/ITO substrates. The height (*h*) of 1 ML can be estimated as *h* = [*M*/(*N*_*A*_ *·ρ*)]^1/3^, where *M*, *N*_*A*_, and ρ represent the molar mass, Avogadro’s constant, and mass density, respectively. In this work, we used 50 and 100 ML. The estimated heights of 1 ML for each IL are as follows: [C_8_C_1_im]­[OTf], *h* = 7.8 Å; [C_2_C_1_im]­[OTf], *h* = 6.8 Å. Thin films composed of pure ILs or mixtures of the two ILs were formed using a simultaneous deposition of the ILs using a total deposition rate of *φ*_*s*_ = (0.6 ± 0.1) Å/s, with the deposition time monitored to achieve the required final thickness. Thin IL films of mixtures with varying compositions of ILs were obtained by adjusting the evaporation temperature of each Knudsen cell to achieve the desirable *φ*_*s*_ for each IL. The condition {*φ*_*s*_ ([C_8_C_1_im]­[OTf]) + *φ*_*s*_ ([C_2_C_1_im]­[OTf]) = (0.6 ± 0.1) Å/s} was maintained for all experiments, and the mole fraction composition of each film was derived assuming molar volume additivity in the ILs mixture. In all experiments, the temperature of the substrates was controlled and kept at *T* = 283 K. Experimental details regarding the formation of thin films of IL mixtures are presented in Table S3 of the Supporting Information.

### Deposition of Rubrene Films by IL-Assisted Vapor Deposition

To explore the capability of the IL films to behave as solvents in vacuum and promote efficient crystallization of organic films, rubrene was thermally evaporated onto the surfaces of ITO and Au/ITO, as well as onto the same substrates precoated with pure [C_2_C_1_im]­[OTf], pure [C_8_C_1_im]­[OTf], and on a [C_2_C_1_im]­[OTf]:[C_8_C_1_im]­[OTf] mixture film (see Figure 2B). Rubrene films with 20 and 200 nm thickness were fabricated by thermally evaporating the organic compound at *T* = 503 K and depositing it onto the substrates, maintaining a constant deposition rate of φ_s_ = 0.3 Å/s. The deposition experiments were performed on substrates maintained at *T* = 283 K. Thin films of IL and rubrene were stored in a desiccator to prevent moisture contamination. Structural and morphological characterization was conducted approximately 1 week after deposition, with the samples maintained in a desiccator during this period.

### Characterization Methods. Scanning Electron Microscopy (SEM)

The morphology of the IL films was examined using scanning electron microscopy (SEM, Hitachi, FlexSEM 1000). Topographic images were acquired at different magnifications (800×, 1600×, 4000×) to investigate the substrate surface and the presence of micro- and nanodroplets or coalesced films on different surfaces. Detailed micrographs were obtained using a backscattered electron (BSE) detector with an acceleration voltage of 3 kV for samples deposited on Au/ITO and 5 kV for samples deposited on Ag/ITO. An in-lens detector was used with a working distance of approximately 6 mm. All SEM parameters were carefully controlled to minimize the electron beam’s potential impact on the IL films’ surface and morphology. The micrographs were further analyzed using image processing software, specifically the *ImageJ*.^64^

A higher-resolution SEM instrument, the FEI Quanta 400 FEG ESEM, was used to investigate the morphology of rubrene films grown onto the IL films. Topographic images were acquired at magnifications of 1000×, 10000×, and 25000×. The micrographs were captured using a secondary electron (SE) detector positioned at a working distance of 13 mm and an accelerating voltage of 15 kV.

### X-ray Photoelectron Spectroscopy (XPS)

X-ray photoelectron spectroscopy (XPS) measurements were performed using an XPS spectrometer (ESCALAB 250Xi, Thermo Scientific) equipped with a monochromatic Al–Kα X-ray radiation source (1486.6 eV) operated at power 200 W. The X-ray spot at the sample surface was 650 μm. The photoelectron spectra were collected at the takeoff angle of 90° with respect to the sample surface by means of a hemispherical electron energy analyzer operated in the constant analyzer energy lens mode (CAE). The pass energy of 150 and 40 eV was used for the survey and high-resolution spectra, respectively. The spectra were accumulated with energy steps of 1 eV for the survey spectra and 0.1 eV for the high-resolution spectra. The high-resolution XPS spectra were fitted using the Gaussian–Lorentzian line shape and Shirley-like background. High-resolution spectra were collected for C 1s, O 1s, N 1s, F 1s, S 2p, In 3d, Sn 3d, and Au 4f. All spectra were processed with *CasaXPS* software (version 2.3, Casa Software Ltd.),^65^ and relative sensitivity factors were obtained from the Kratos Library.

### X-ray Diffraction (XRD)

The X-ray diffraction patterns for rubrene films deposited on ITO and Au surfaces and those grown on ILs were acquired at room temperature using a Rigaku Smartlab diffractometer. This system features a copper Kα X-ray source (wavelengths 1.54059 Å and 1.54441 Å) with a rotating anode tube operating at 6.75 kW (45 kV, 150 mA). The instrument was configured in the Bragg–Brentano geometry, with coupled source and detector movements and a fixed sample holder. Data collection was performed using the θ/2θ scanning mode, covering an angular range from 5° to 60° with a step size of 0.005 degrees and a scanning speed of 5 degrees per minute. A knife edge was positioned approximately 1.5 mm above the sample to mitigate air-scattering effects. A nickel filter was also placed after the sample to eliminate Kβ radiation. The setup included a pair of incident and receiving slits, each set at 5 degrees, located before and after the sample. Two confinement slits, one horizontal and one vertical, were used to create an illuminated area on the sample of approximately 2 mm × 2 mm.

## Results and Discussion

### Thin Films of Ionic Liquid Mixtures

Figure 3 illustrates the distinct morphologies of [C_2_C_1_im]­[OTf] and [C_8_C_1_im]­[OTf] films co-deposited on ITO for varying ratios of the two ILs and the two used thicknesses (100 and 50 ML in the IL mixture). Upon examining the SEM images of the 100 ML films (Figures 3a to 3e), it is noticeable that when only [C_2_C_1_im]­[OTf] is present (Figure 3a), there is an abundance of small, highly spherical droplets. Conversely, when only [C_8_C_1_im]­[OTf] is present (Figure 3e), the droplets are larger and more irregularly shaped, consistent with previous studies on pure ILs.^13,14,34^ Observing Figures 3a to 3c, it becomes evident that as the amount of [C_8_C_1_im]­[OTf] increases, the droplet size also increases, resulting in larger and more irregular shapes. For mixtures with a thickness of 50 ML (Figures 3f to 3j), the smaller amount deposited leads indeed to less coalescence than observed in the 100 ML samples, and smaller droplets are seen with more uniform sizes. However, it can also be concluded that an increase in the mole fraction of [C_8_C_1_im]­[OTf] in the resulting film still results in larger droplet sizes. The formation of larger droplets in IL mixture films with a higher proportion of [C_8_C_1_im]­[OTf] compared to [C_2_C_1_im]­[OTf] may also result from the lower surface tension (see Table S1 of the Supporting Information) of [C_8_C_1_im]­[OTf]. This behavior is characteristic of alkylimidazolium-based ILs with longer alkyl chains.^13,17^

**Figure 3:**
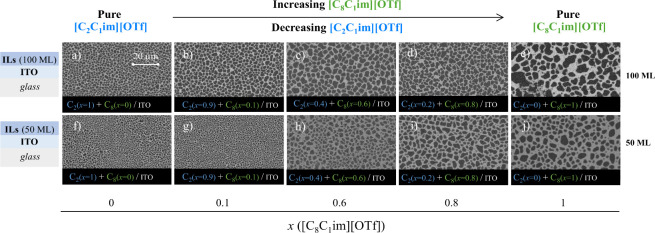
Morphology of thin films composed of mixtures of [C_2_C_1_im]­[OTf] and [C_8_C_1_im]­[OTf], deposited on ITO-coated glass surfaces by simultaneous deposition of both ILs, with a varying mole fraction (*x*) of each IL in the resulting film: *x*{[C_8_C_1_im]­[OTf]} = 0 (images a and f); *x*{[C_8_C_1_im]­[OTf]} = 0.1 (images b and g); *x*{[C_8_C_1_im]­[OTf]} = 0.6 (images c and h); *x*{[C_8_C_1_im]­[OTf]} = 0.8 (images d and i); *x*{[C_8_C_1_im]­[OTf]} = 1 (images e and j). Top views acquired by SEM using a backscattered electron detector. These films theoretically consist of 100 ML (images a–e) or 50 ML (images f–j).

Subsequently, the co-evaporation process of the studied ILs was performed using substrates with different surfaces: ITO, Ag/ITO, and Au/ITO (see substrate schematics at the top of Figure 2). This allowed for distinguishing the morphology of the mixtures on these distinct surfaces and assessing the impact of the interface on the film’s structure. Figure 4 presents SEM images illustrating the corresponding morphologies for 100 ML deposited on each substrate.

**Figure 4:**
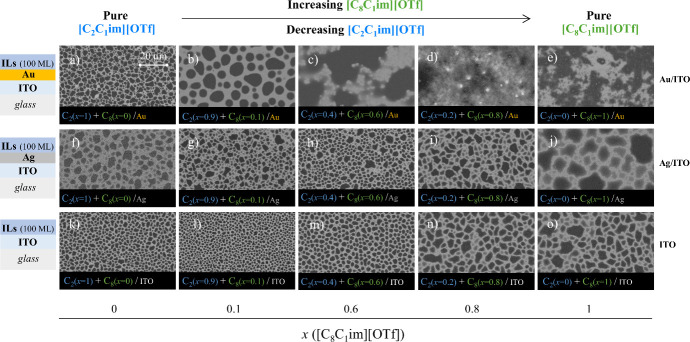
Morphology of thin films composed of mixtures of [C_2_C_1_im]­[OTf] and [C_8_C_1_im]­[OTf], deposited on Au/ITO/glass (images a–e), Ag/ITO/glass (images f–j) and ITO/glass (images k–o) surfaces by simultaneous deposition of both ILs, with a varying mole fraction (*x*) of each IL in the resulting film: *x*{[C_8_C_1_im]­[OTf]} = 0 (images a, f, and k); *x*{[C_8_C_1_im]­[OTf]} = 0.1 (images b, g, and l); *x*{[C_8_C_1_im]­[OTf]} = 0.6 (images c, h, and m); *x*{[C_8_C_1_im]­[OTf]} = 0.8 (images d, i, and n); *x*{[C_8_C_1_im]­[OTf]} = 1 (images e, j, and o). Top views acquired by SEM using a backscattered electron detector. These films theoretically consist of 100 ML.

Figures 4a to 4e depict the film’s morphology on the Au surface (top images), Figures 4f to 4j on the Ag surface (middle images), and Figures 4k to 4o on the ITO surface (bottom images). There are some differences when comparing the images at the top of Figure 3 (3a to 3e) with those at the bottom of Figure 4 (4k to 4o). These images theoretically should correspond to the same morphology, as they both relate to the deposition of 100 ML on ITO. It is important to note that the results in Figure 3 represent the deposition of IL on a surface consisting solely of an ITO substrate, whereas those in Figure 4 correspond to deposition on an ITO substrate that was partially covered with metal. The images in the third column of Figure 4 (4k to 4o) specifically illustrate the morphology of the IL on the ITO side. Achieving perfect reproducibility in these results is inherently challenging, as even small variations in deposition parameters can influence the nucleation and growth of the film. Furthermore, the process of coating half of the substrate with metal involved masking an ITO portion with Kapton tape, followed by sputtering and the subsequent removal of the tape, which could impact wettability. Despite these factors, both Figures 3 and 4) exhibit the same overall trend: the formation of larger droplets with increasing mole fraction of [C_8_C_1_im]­[OTf] in the mixture. For the Ag/ITO and ITO surfaces, it is evident from the images that as the proportion of [C_8_C_1_im]­[OTf] increases in the mixture, there is an enlargement in the droplet size. The droplets display irregular shapes, particularly on the Ag surface, suggesting slow peripheral diffusion of IL along their edges. This may suggest a stronger affinity between the ILs and Ag, as the droplets tend to spread out more on the surface. Nevertheless, the results indicate that the IL mixture deposited on both ITO and Ag surfaces led to the formation of micro- and nanodroplets, even with an increased quantity of long-chain IL. Regarding Ag substrates, the formation of a 2D layer (not detectable by SEM) cannot be dismissed, as it is known that on many surfaces, the ILs tend to form initially an ultrathin film, followed by island growth.^38^ Figure 5 presents graphical representations illustrating the influence of film composition on the properties of IL films formed by the co-evaporation of [C_2_C_1_im]­[OTf] and [C_8_C_1_im]­[OTf] on ITO surfaces (Graphs a and b) and Ag surfaces (Graphs c and d). These data provide insights into the impact of the proportion of each IL in the mixture on two key parameters characterizing the IL droplets: surface coverage (Graphs 5a and 5c) and modal diameter (Graphs 5b and 5d). For 100 ML deposited on ITO, surface coverage appears to increase slightly only when the mixture is predominantly composed of [C_8_C_1_im]­[OTf], as shown in Figure 5a. When [C_2_C_1_im]­[OTf] predominates in the mixture, surface coverage is high due to the formation of numerous smaller droplets. However, as the content of [C_8_C_1_im]­[OTf] increases and becomes predominant, several factors contribute to maintaining the surface coverage. First, forming larger droplets with lower contact angles enhances their spreading on the surface, leading to increased coverage. Second, small nanodroplets forming between the larger ones fill the empty spaces, further contributing to the overall surface coverage. Additionally, it is evident that for a smaller deposition amount, such as 50 ML, the surface coverage is smaller as compared to samples with a deposition of 100 ML. Observing Figure 5b, it becomes clear that the modal diameter of the droplets increases as the amount of [C_8_C_1_im]­[OTf] in the mixture increases. This indicates that [C_8_C_1_im]­[OTf] promotes the formation of larger droplets. Coalescence processes are more pronounced with a higher proportion of [C_8_C_1_im]­[OTf], resulting in the formation of larger droplets in the deposited film. As expected, an increase in the amount of IL deposited led to a considerable enlargement of droplet size (higher modal diameters). According to the results depicted in Figure 5d, as the content of [C_8_C_1_im]­[OTf] increases in the mixture, the diameter of the droplets on both Ag and ITO surfaces also increases.

**Figure 5:**
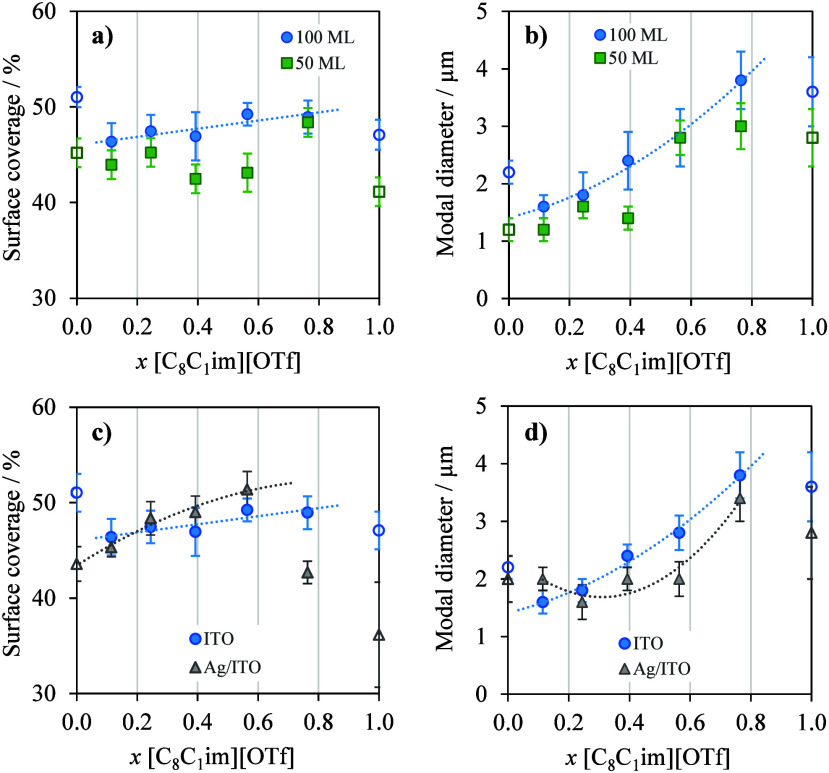
Influence of film composition on droplet properties formed by co-evaporation of [C_2_C_1_im]­[OTf] and [C_8_C_1_im]­[OTf]. Surface coverage (graphs a and c) and modal diameter (graphs b and d) as a function of the mole fraction of [C_8_C_1_im]­[OTf]. Plots for IL droplets deposited on ITO at different amounts: 50 ML (solid squares) and 100 ML (solid circles) (graphs a and b). Plots for IL (100 ML) droplets deposited on both ITO (solid circles) and Ag/ITO surfaces (solid triangles) (graphs c and d).

Furthermore, it can be concluded that the modal diameter of the droplets formed on Ag surfaces is slightly smaller than those on ITO; however, this results in higher surface coverage, as observed in Figure 5c. When the mole fraction of [C_8_C_1_im]­[OTf] is significantly higher than that of [C_2_C_1_im]­[OTf], the surface coverage of the droplets decreases markedly (Figure 5c). In such cases, some areas of the Ag surface may likely be covered by a coalesced IL film. Figure 5 also shows that in all the graphs, the values corresponding to the pure ILs appear as outliers compared to the observed trends for the mixtures. These findings demonstrate the distinct behavior between films composed of pure ILs and those formed by IL mixtures. Thin films of pure ILs, consisting solely of [C_2_C_1_im]­[OTf] or [C_8_C_1_im]­[OTf], are composed of uniform ion pairs, resulting in consistent and predictable intermolecular forces. However, when mixing the ILs, the presence of the two cations (C_2_C_1_im and C_8_C_1_im) leads to varied intermolecular interactions. These new interactions affect the physical properties of the mixture, leading to variations in droplet nucleation, growth dynamics, and overall morphology during thermal evaporation. Consequently, the number of droplets, their modal diameter, and surface coverage differ significantly as compared to films formed from pure ILs.

It is noteworthy that the results obtained on the Au/ITO surface (Figures 4a to 4e) differ from those observed on the Ag/ITO and ITO surfaces. The Au/ITO surface is particularly relevant in this study because previous findings suggest that long-chain ILs completely wet Au surfaces, leading to consistent layer-by-layer growth of ILs.^13,14^ Figures S8–S11 of the Supporting Information present additional details regarding the morphological characterization of these films. Figures S8 and S9 present SEM and optical microscope images for the various IL compositions in the IL mixture film. The optical microscope, operating in brightfield transmission mode, was used for the initial assessment of film morphology immediately after the deposition process. Analysis of the optical images revealed a consistent change in morphology as the content of [C_8_C_1_im]­[OTf] increased. The SEM images provided significantly greater detail of the surfaces, but the main conclusions drawn from both techniques remained consistent, showing a progressive increase in wettability with the mole fraction of [C_8_C_1_im]­[OTf]. As a main observation, an increase in [C_8_C_1_im]­[OTf] concentration in the mixture leads to enhanced coalescence (Figures 4a–4e). This coalescence is notably more pronounced than that observed on both ITO and Ag surfaces. A continuous film formation becomes evident when the amount of [C_8_C_1_im]­[OTf] exceeds that of [C_2_C_1_im]­[OTf]. Even when [C_2_C_1_im]­[OTf] is present in larger amounts (as seen in images 4a and 4b), the film exhibits superior spreading behavior, forming larger droplets with lower sphericity compared to ITO. This observation provides further evidence of the favorable wetting ability of ILs on Au surfaces. When [C_8_C_1_im]­[OTf] dominates the mixture, the film exhibits extensive spreading, leading to the formation of an almost completely coalesced film. Images 4c, 4d, and 4e show very large microstructures with low contact angles alongside empty spaces within the film. This phenomenon is likely attributed to an insufficient amount of material deposited, which may not have been enough to form a fully coalesced film. Based on these findings, it is clear that IL mixture films exhibit a strong affinity for Au surfaces, particularly at higher [C_8_C_1_im]­[OTf] concentrations. There is probably low interfacial tension in those cases, indicating a better affinity between the IL and the Au substrate.^13,14,31,37–39^ In principle, this behavior can be attributed to the exceptional adsorption of long-chain imidazolium-based ILs onto Au substrates.^37–39^ This initial adsorption facilitates the improved spreading of subsequent MLs due to dispersive interactions among the alkyl chains, contributing to layer-by-layer growth.

Although all the films were stored in a desiccator, potential changes in their morphology over time cannot be entirely ruled out. Nevertheless, we have concluded that, at these thicknesses, the morphology of the films remains stable. In a previous publication, we conducted a time-dependent study of the film morphology of [C_2_C_1_im]­[OTf] on ITO/glass.^36^ The results indicated no apparent influence of time on the morphology of the IL film (specifically for 50 and 150 ML thicknesses). In this work, we also concluded that the morphology of the IL mixture film did not change significantly over time (Figures S10 and S11). At the mesoscale, for [NTf_2_]- or [OTf]-based ILs, we have systematically concluded that the film’s morphology remains relatively stable once the deposition is completed.

The results presented offer insight into the morphology of IL mixture films on substrates featuring sections of ITO/glass and metal/ITO/glass. The morphologies presented so far were obtained far from the interface between the two surfaces. However, to assess the importance of the interface, the morphology of IL mixture films near the interface was specifically examined and captured at the junction between the two surfaces. Figure 6 illustrates detailed SEM images, with the top images (Figures 6a to 6e) depicting the morphology of the films near the ITO/Ag interface and the bottom images (Figures 6f to 6j) depicting the morphology of the films near the ITO/Au interface. The ILs exhibit enhanced mobility near the interface, with ion pairs tending to migrate from ITO to the metal. This behavior is driven by the ILs’ inherent affinity for metallic surfaces. The possibility of metal atoms being deposited on the ITO region near the boundary due to deflected metal atoms during the sputtering process is an important factor that may explain this behavior. Such a phenomenon could indeed influence the wetting behavior and morphology of the ILs in these regions.

**Figure 6:**
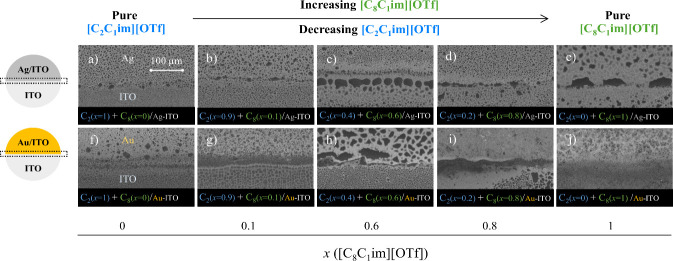
Morphology of thin films near the metal-ITO interface. These films consist of mixtures of [C_2_C_1_im]­[OTf] and [C_8_C_1_im]­[OTf], deposited on Ag/ITO/glass (images a–e) and on Au/ITO/glass (images f–j) surfaces by simultaneous deposition of both ILs, with a varying mole fraction (*x*) of each IL in the resulting film: *x*{[C_8_C_1_im]­[OTf]} = 0 (images a and f); *x*{[C_8_C_1_im]­[OTf]} = 0.1 (images b and g); *x*{[C_8_C_1_im]­[OTf]} = 0.6 (images c and h); *x*{[C_8_C_1_im]­[OTf]} = 0.8 (images d and i); *x*{[C_8_C_1_im]­[OTf]} = 1 (images e and j). Top views acquired by SEM using a backscattered electron detector. These films theoretically consist of 100 ML.

In addition, due to the greater affinity of ILs with larger alkyl chains, an increase in [C_8_C_1_im]­[OTf] concentration in the mixture leads to the formation of larger droplets near the interface on ITO. This effect is attributed to the enhanced mobility of the ILs, which promotes coalescence. Moreover, this behavior is more pronounced at the Au interface, as ILs exhibit a stronger affinity for Au compared to Ag. Thus, this analysis clearly illustrates that the presence of a metallic surface significantly influences the morphology of ILs at the ITO interface. Notably, the morphology of the IL film in the interface regions was found to remain stable. In fact, we obtained experimental results demonstrating that the morphology of the IL film remains unchanged even at the interface between ITO and Ag/ITO. Figure 7 shows the morphology of [C_2_C_1_im]­[OTf] near the Ag-ITO interface, as observed by SEM from the same sample at different time points. The similarity of the images also highlights the negligible effect of the electron beam on these samples.

**Figure 7:**
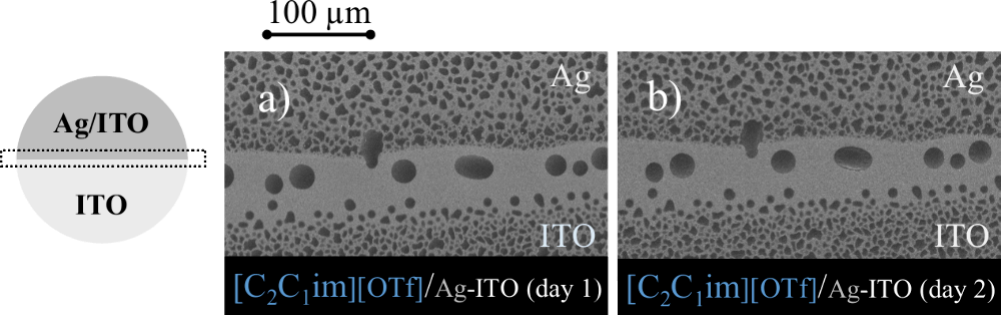
Morphology of thin films of [C_2_C_1_im]­[OTf] near the Ag-ITO interface. Top views acquired by SEM using a backscattered electron detector. Micrographs were obtained from the same spot on the sample after one (image a) and two (image b) days of deposition. These films theoretically consist of 150 ML.

Subsequently, XPS analysis was conducted to verify the surface composition and integrity of the IL mixture films. Figure 8 displays the XPS spectra of IL films, each with 100 ML thickness, deposited on Au surfaces. The overall XPS results, including data for the substrates and ILs deposited on ITO, are provided in Figures S14–S32 of the Supporting Information. Before XPS analysis, all samples were stored in an argon atmosphere to minimize contamination. However, completely eliminating carbon contamination is extremely challenging, as these substrates inherently contain adventitious carbon. Additionally, XPS analysis itself can introduce contamination. The data from the substrates reveal the presence of adventitious carbon–particularly on ITO/glass, where XPS analysis detects carbon species covering approximately 30–40% of the surface. The C 1s spectrum confirms adventitious carbon contamination, with detected C–C, C–O, and C=O components (Figure S14). However, the thickness of this contamination layer is estimated at most 1–2 nm. In contrast, carbon contamination on metal surfaces is lower, typically below 20% in the case of Au. Figures S15 and S17 show the XPS survey spectra of the ITO/glass and Ag/ITO/glass surfaces after the removal of adventitious carbon by argon sputtering. In our deposition experiments, such cleaning was not possible, but exposure of the substrates to air was always minimized. Surface contamination can significantly influence the growth of IL films. Nanoscale studies must be conducted using well-defined solid substrates, as even trace amounts of carbon can affect the nucleation and growth of the first monolayer. However, our focus has been on thick IL coatings^13,14,18,33–36,44,48^ rather than the initial monolayer adsorption, where adventitious carbon has a more pronounced impact. While it is important to acknowledge that all substrates inevitably contain some surface contamination, they are typically used in this state for practical applications. The key is to minimize contamination as much as possible to mitigate its influence on the conclusions drawn from the experimental data.

**Figure 8:**
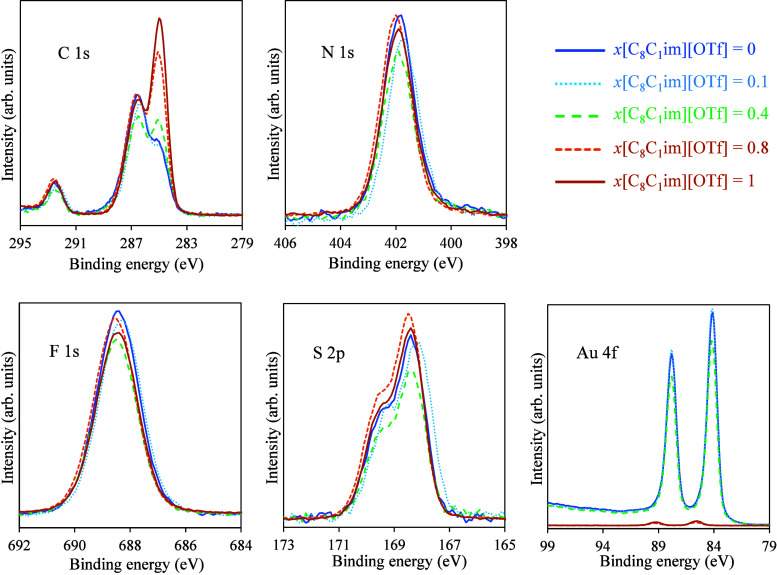
High-resolution XPS spectra of IL films deposited on Au surfaces by simultaneous deposition of [C_2_C_1_im]­[OTf] and [C_8_C_1_im]­[OTf], with a varying mole fraction (*x*) of each IL in the resulting film: *x*{[C_8_C_1_im]­[OTf]} = 0 (blue solid curve); *x*{[C_8_C_1_im]­[OTf]} = 0.1 (blue dashed curve); *x*{[C_8_C_1_im]­[OTf]} = 0.4 (green dashed curve); *x*{[C_8_C_1_im]­[OTf]} = 0.8 (dashed brown curve); *x*{[C_8_C_1_im]­[OTf]} = 1 (solid brown curve). The data were acquired for C 1s, N 1s, F 1s, S 2p, and Au 4f.

XPS spectra were collected for pure ILs, [C_2_C_1_im]­[OTf] and [C_8_C_1_im]­[OTf], as well as for their mixtures at varying mole ratios of each IL: mixture with *x*[C_8_C_1_im]­[OTf] = 0.1; mixture with *x*[C_8_C_1_im]­[OTf] = 0.4; mixture with *x*[C_8_C_1_im]­[OTf] = 0.8. Multiple peaks can be observed when analyzing the C 1s spectrum, indicating the presence of different carbon environments in the IL films. The primary peak around 285 eV is typically associated with C–C bonds, while the subsequent peak around 286 eV is associated with C–N bonds. These peaks correspond to the alkylimidazolium cation. Additionally, in samples where part of the substrate is exposed, the intensity of the peaks in the C 1s spectrum is influenced by adventitious carbon contamination. This effect is more pronounced in IL mixtures deposited on ITO, as a larger percentage of the substrate remains uncovered by the IL. A peak at higher energy, around 294 eV, is typically from CF_3_ groups and is related to the anion. It is observed that the peak attributed to the C–C bonds increases in intensity with the increasing proportion of [C_8_C_1_im]­[OTf] in the mixture. In contrast, the peak attributed to the anion remains constant. The N 1s spectrum shows a single prominent peak around 402 eV, corresponding to nitrogen in the imidazolium ring. As expected, an increase in the mole fraction of [C_8_C_1_im]­[OTf] in the mixture led to an increase in the {C_cation_/C_anion_} and {C_cation_/N_cation_} ratios, as derived from the XPS results (Table S2 of the Supporting Information). This conclusion is more pronounced on the Au surface, as, in the case of ITO, the ratios are influenced by the information provided by the exposed substrate. The F 1s spectrum exhibits a peak around 688 eV, indicating fluorine atoms from the triflate (OTf) anion. The S 2p spectrum shows a doublet with peaks around 168–169 eV, which is characteristic of sulfur in the OTf anion. In the Au 4f spectrum, peaks specific to Au are only visible in mixtures with a higher proportion of [C_2_C_1_im]­[OTf]. This aligns with the SEM observations, which indicate the formation of droplets while leaving a significant portion of the substrate exposed (Figures 4a and 4b). In contrast, for pure [C_8_C_1_im]­[OTf] and for mixtures with a higher [C_8_C_1_im]­[OTf] mole fraction (0.8), the Au signal is significantly reduced. This also corresponds to the SEM results, which show nearly complete wetting of the substrate by the ILs, leaving no exposed Au surface (Figures 4d and 4e).

### Ionic Liquid-Assisted Vapor Deposition of Rubrene

This section discusses the process and outcomes of IL-assisted vapor deposition of rubrene, with the main goal of using ILs to serve as solvents under vacuum to enhance the properties of the resulting rubrene films. The IL films were deposited on ITO/glass and Au/ITO/glass substrates. Specifically, thin films of [C_2_C_1_im]­[OTf], [C_8_C_1_im]­[OTf], and a mixture of [C_2_C_1_im]­[OTf] (*x* = 0.6) and [C_8_C_1_im]­[OTf] (*x* = 0.4) were investigated. The SEM images of these films are shown in Figure S12 of the Supporting Information. On the ITO surface, it is evident that [C_2_C_1_im]­[OTf] forms smaller droplets with higher sphericity, whereas [C_8_C_1_im]­[OTf] produces larger and more irregularly shaped droplets. The mixture displays characteristics that are intermediate between these two extremes. Upon deposition onto Au, the [C_2_C_1_im]­[OTf] droplets appear larger, while [C_8_C_1_im]­[OTf] forms a nearly fully coalesced film. This observation, consistent with the previous results of this work, underscores the stronger affinity of ILs for the Au surface. Subsequently, rubrene was deposited at thicknesses of 20 nm (Figure 9) and 200 nm (Figure 10) onto films of pure ILs and their mixtures on ITO and Au surfaces. Rubrene was also deposited directly onto clean ITO and Au surfaces for comparison. Rubrene deposited on ITO (Figures 9a and 10a) appears as an amorphous film, whereas microcrystals are formed on the Au surface (Figures 9b and 10b). The difference in rubrene morphology on ITO versus Au surfaces can be attributed to several factors. First, the interaction between rubrene molecules and the substrate affects nucleation and crystal growth. Au surfaces may promote stronger interactions, leading to the formation of microcrystals. In addition, Au typically has a lower surface energy than ITO, which can facilitate the ordering of molecules into a crystalline structure. When rubrene was deposited onto substrates coated with droplets of [C_2_C_1_im]­[OTf], its crystallinity showed no apparent improvement. The SEM images suggest that rubrene forms amorphous structures between the IL droplets. Rubrene molecules directly deposited onto the droplets are likely dissolved, followed by recrystallization. Visible rubrene aggregates cover the IL droplets (Figure 9c). For larger thicknesses, rubrene appears to begin forming a continuous film across the entire surface, as suggested by the presence of holes at the centers of the droplets (Figure 10c). On Au surfaces, a different growth mechanism is observed. Due to the good affinity of rubrene for Au, there is a higher tendency for rubrene films to grow in areas without IL (Figures 9d and 10d). Further, the short-chain IL was found to be a weak liquid medium for the crystallization of rubrene, as it is clear that the deposition of rubrene did not significantly alter the size of the [C_2_C_1_im]­[OTf] droplets. Even with increased deposition, there was no substantial change in droplet shape, particularly on Au surfaces, as seen when comparing Figures 9d and 10d. Different results are observed with rubrene deposition on [C_8_C_1_im]­[OTf] droplets. First, there is a complete change in the morphology of the IL film on ITO/glass, which appears now fully coalesced (Figures 9e and 10e). This experimental result was recently observed with a similar long-chain IL, [C_8_C_1_im]­[NTf_2_], used as a liquid medium for the formation of crystalline perylene films.^18^ This is unsurprising as long-chain ILs have a good affinity for organic compounds. As a result, both perylene and rubrene molecules are more soluble in long-chain imidazolium-based ILs than in short-chain ILs.

**Figure 9:**
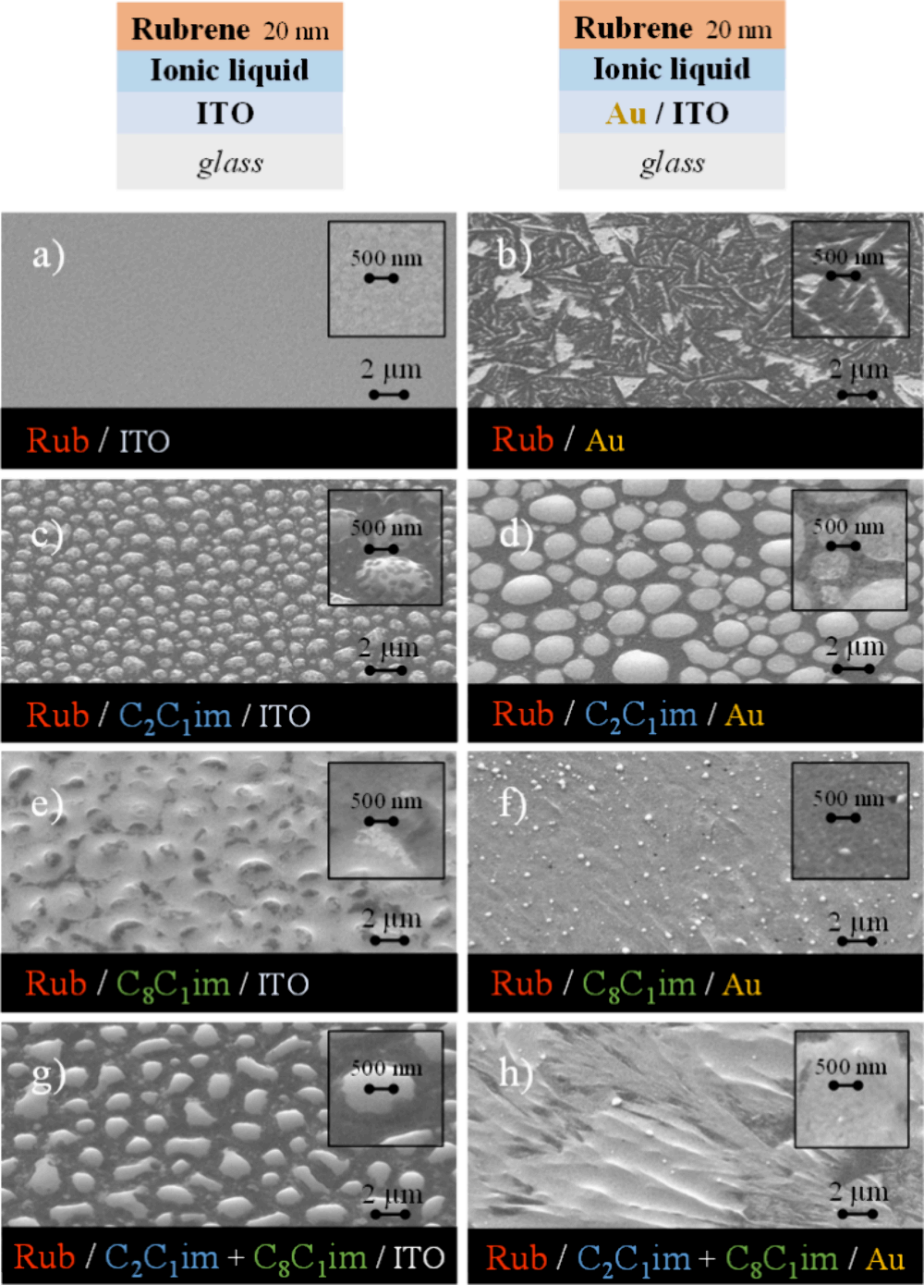
Morphology of rubrene (Rub) films (20 nm) deposited on ITO (a) and Au (b). Rubrene deposited on ITO (images on the left) and on Au (images on the right) surfaces coated with [C_2_C_1_im]­[OTf] (c and d), [C_8_C_1_im]­[OTf] (e and f), and mixtures of [C_2_C_1_im]­[OTf] (*x* = 0.6) and [C_8_C_1_im]­[OTf] (*x* = 0.4) (g and h). Lateral views at 45° acquired through high-resolution SEM by using a secondary electrons detector.

**Figure 10:**
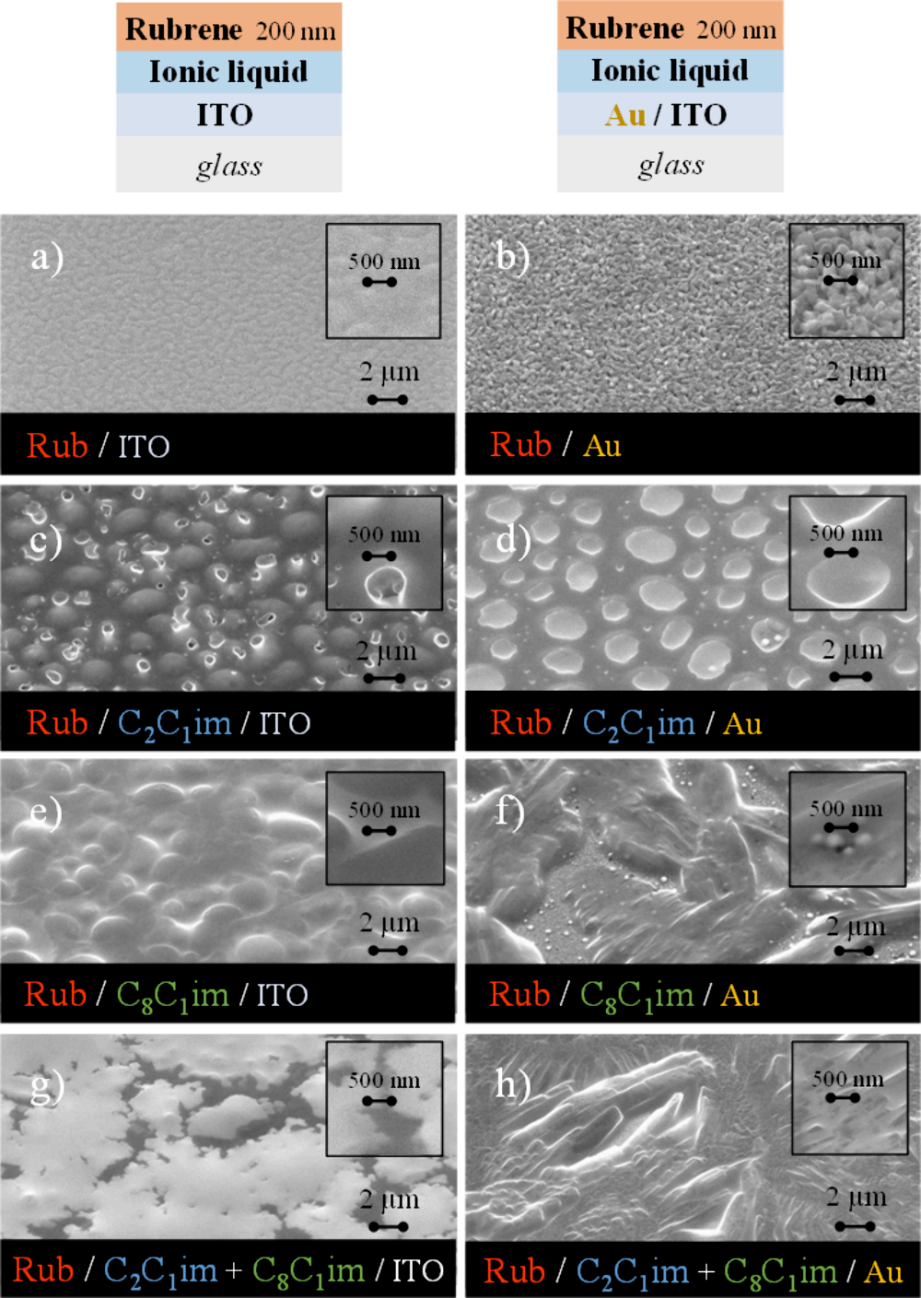
Morphology of rubrene (Rub) films (200 nm) deposited on ITO (a) and Au (b). Rubrene deposited on ITO (images on the left) and on Au (images on the right) surfaces coated with [C_2_C_1_im]­[OTf] (c and d), [C_8_C_1_im]­[OTf] (e and f), and mixtures of [C_2_C_1_im]­[OTf] (*x* = 0.6) and [C_8_C_1_im]­[OTf] (*x* = 0.4) (g and h). Lateral views at 45° acquired through high-resolution SEM by using a secondary electrons detector.

The experimental results indicate that the underlying substrate strongly influences the rubrene morphology and crystallinity. Compared to ITO, the crystallinity of rubrene appears to be enhanced on Au surfaces. Even with a 20 nm rubrene film, rubrene appears homogeneously distributed across the entire surface, and its crystallinity seems to be improved (Figure 9f). Increasing the deposition thickness to 200 nm significantly enhances the film crystallinity, with visibly larger rubrene microstructures being formed (Figure 10f). The mixture of [C_2_C_1_im]­[OTf] and [C_8_C_1_im]­[OTf] appears to drive the growth of rubrene films with seemingly highly crystalline morphology. Figures 9h and 10h highlight the clear formation of very large crystals growing horizontally onto Au. Detailed images are presented in Figure S13 of the Supporting Information. On the ITO substrate, the IL mixture induced the formation of a different morphology of the organic compound, which is likely crystalline as well (Figure 10g). The IL-assisted vapor deposition process allows for the formation of crystalline organic films through successive processes of dissolution of amorphous material followed by recrystallization.^48^

There are some reports in the literature concerning ionic liquid/rubrene single-crystal interfaces.^21,66^ According to Horike et al., rubrene molecules initially nucleate within IL microdroplets, forming rubrene-concentrated IL domains.^21^ As rubrene crystallites grow, they appear to displace or “shed″ the IL microdroplets. Once the crystallites become larger than the microdroplets, the ILs may instead surround the crystal perimeter, acting as additional nucleation sites. This suggests that while rubrene initially interacts with and may partially replace IL in localized regions, some IL likely remains at the crystal periphery. Ultimately, the presence of IL at the interface influences the growth dynamics, leading to two-dimensional crystal formation constrained by the IL layer thickness. Furthermore, the same study demonstrated that rubrene deposited directly onto the substrate without ILs remained amorphous, which aligns with our findings. In our work, we set the substrate temperature at 283 K to deposit both the IL and rubrene. Other studies have explored depositions at higher temperatures (up to 373 K).^67^ Such differences in substrate temperature can significantly influence the morphology and crystallinity of rubrene films. Higher substrate temperatures can potentially promote the formation of larger grains. The results presented herein demonstrate that rubrene can crystallize easily at room temperature using IL-assisted vapor deposition; therefore, even better results might be expected at higher substrate temperatures.

Our experimental results suggest that IL mixtures may enhance rubrene crystallization, with this effect being further amplified on the Au substrate. This improvement might be explained by the enhanced solubility of rubrene in the mixed ILs, which facilitates the dissolution and recrystallization processes by providing a medium where rubrene molecules can undergo increased molecular diffusion and reorganization. The presence of both short- and long-chain ILs in the mixture likely creates an optimal environment for crystal growth by balancing the interfacial properties of ILs, as well as the solubility and mobility of rubrene. Experimentally, it was not possible to determine the saturation solubility of rubrene in the studied ILs. However, there might not be a direct correlation between macroscopic solubilities and the systems studied in this work, which involve surfaces that are not entirely covered by ILs. In particular, the ILs form micro- and nanodroplets acting as local solvents in the vacuum environment, complicating the relationship between bulk and surface solubility. There are no reports on the saturation solubility of rubrene in ILs, even in prior studies on IL-assisted vapor deposition of rubrene. However, some studies have reported rubrene solubility in molecular solvents, which can provide helpful context.^68,69^ The solubility of rubrene in toluene, *p*-xylene, and 1-propanol has been reported as 0.03, 0.01, and 0.0001 mol/L, respectively, highlighting a higher solubility of rubrene in nonpolar solvents.^68,69^ It is reasonable to assume that [C_8_C_1_im]­[OTf], due to its longer alkyl chain, may dissolve rubrene differently compared to [C_2_C_1_im]­[OTf]. The longer alkyl chain in [C_8_C_1_im]­[OTf] may enhance interactions with rubrene, facilitating its solubilization. Additionally, the solubility of rubrene in ILs is likely influenced by the interactions between the ILs and the substrate surface. The formation of IL micro- and nanodroplets on the surface could alter the local concentration of rubrene and affect its effective solubility. We have estimated that the deposition of 20 nm (≈ 5·10^–5^ mol/m^2^) and 200 nm (≈ 5·10^–4^ mol/m^2^) of rubrene on 100 ML of IL film corresponds to nominal concentrations of ≈ 0.6 mol/dm^3^ and ≈ 5.8 mol/dm^3^, respectively. For comparison, a 1 nm deposition of rubrene would correspond to a nominal concentration of ≈ 0.03 mol/dm^3^, suggesting that a substantial amount of rubrene could be solubilized during the early stages of deposition.

UV–vis absorption spectra comparison of rubrene films deposited on various surfaces are presented in Figure S33 of the Supporting Information. The findings highlight that the absorption spectra of rubrene deposited on ITO and ILs show similarities. However, a careful analysis of the results reveals that compared to rubrene dissolved in DCM or a rubrene film deposited on ITO, the highest absorption energy peak appears enhanced for rubrene films deposited on ITO/glass precoated with [C_8_C_1_im]­[OTf] and on ITO/glass precoated with a mixture of [C_2_C_1_im]­[OTf] and [C_8_C_1_im]­[OTf]. This correlates with the higher crystallinity of rubrene observed in these cases.

XRD analysis was performed to gain a more comprehensive understanding of the crystallinity of rubrene films (200 nm) deposited on various surfaces. Figure 11 presents the XRD patterns of rubrene films (Figure 11A) deposited on Au/ITO/glass surfaces, as well as on ITO/glass and Au/ITO/glass substrates previously coated with the pure ILs and their mixtures. Additionally, Figure 11B compares the estimated crystallite sizes of rubrene films grown on the different substrates. The crystallite sizes were determined using two methods: the traditional approach, which applies the Scherrer equation to individual peaks and then averages the results, and the Williamson-Hall (W–H) representation. Further details are provided in the SI. Analyzing Figure 11A, it is evident that the (100) peak in the 2θ range of 6–8° attributed to rubrene is not observed when rubrene is deposited on ITO, confirming its characterization as an amorphous film, as previously shown in the SEM images. A similar behavior was observed for rubrene deposited on Au, although its morphology would suggest some degree of crystallinity. However, only XRD peaks related to the substrates (ITO and Au) were experimentally detected. The XRD pattern of rubrene single crystals is typically characterized by the presence of the (100), (200), (300), (400), (500), (600), and (700) peaks in the 2θ ranges of 6–8°, 12–14°, 19–21°, 26–28°, 32–34°, 40–42°, and 46–48°, respectively (see Figures S34 and S35 of the Supporting Information).^70^ Notably, the characteristic peaks of rubrene crystals were only detected in this work for rubrene films obtained using ILs as substrates. An exception is observed when the organic was deposited on [C_2_C_1_im]­[OTf]/ITO, as no crystallization signals were detected, which agrees with conclusions derived from SEM analysis. With the same IL but using the Au substrate, the diffraction peaks (100), (300), and (600) were identifiable. For rubrene films deposited on [C_8_C_1_im]­[OTf] (on both ITO and Au surfaces), the (100), (200), and (300) peaks were clearly detected. The best results, however, were obtained with mixtures of [C_2_C_1_im]­[OTf] and [C_8_C_1_im]­[OTf]. In these cases, the (100), (200), (300), (400), and (600) diffraction peaks typical of rubrene crystals were clearly observable on both ITO and Au surfaces.

**Figure 11:**
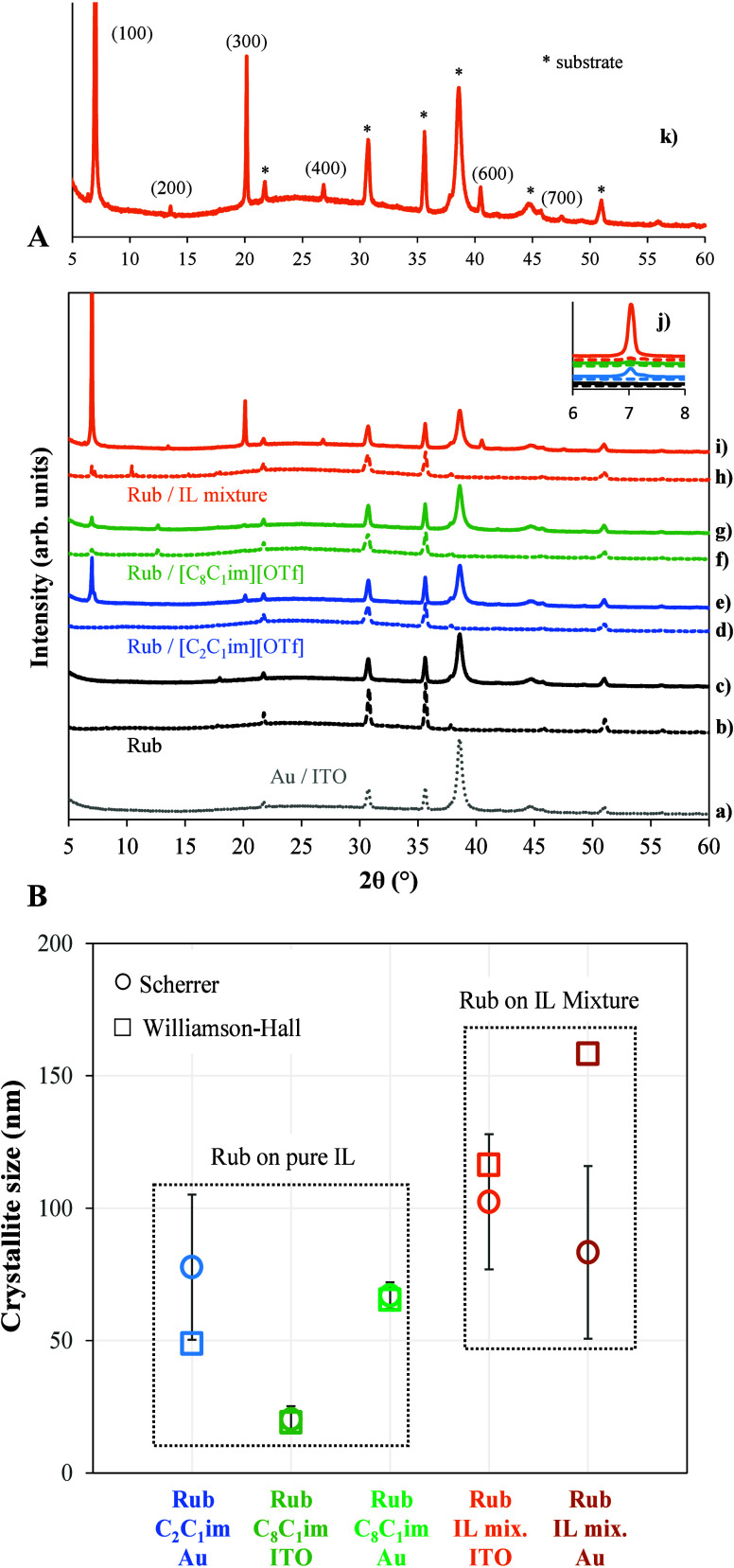
(A) XRD patterns of various rubrene (Rub) films: (a) Au/ITO substrate; (b) rubrene film on ITO/glass; (c) rubrene film on Au/ITO/glass; (d) rubrene film on [C_2_C_1_im]­[OTf]/ITO; (e) rubrene film on [C_2_C_1_im]­[OTf]/Au/ITO; (f) rubrene film on [C_8_C_1_im]­[OTf]/ITO; (g) rubrene film on [C_8_C_1_im]­[OTf]/Au/ITO; (h) rubrene film on a mixture of [C_2_C_1_im]­[OTf] and [C_8_C_1_im]­[OTf] (on ITO); (i) rubrene film on a mixture of [C_2_C_1_im]­[OTf] and [C_8_C_1_im]­[OTf] (on Au/ITO); (j) zoomed-in XRD pattern of the peak (100) in the 2θ range of 6–8°; (k) detailed XRD pattern with Miller indexes for the rubrene film deposited on Au/ITO previously coated with a mixture of [C_2_C_1_im]­[OTf] and [C_8_C_1_im]­[OTf] (spectrum k is the same as spectrum i but with a blown-up intensity scale). (B) Estimated crystallite sizes of rubrene films grown using ionic liquid films.

While pure ILs effectively promote rubrene crystallization, combining short-chain and long-chain ILs significantly enhances its crystallinity. Remarkably, this enhancement is even more pronounced with the use of Au substrates, highlighting the synergistic effect of combining IL mixtures with Au substrates on the structural ordering and crystallinity of the rubrene films formed thereof. SEM images corroborate these findings (Figure 10), demonstrating enhanced crystallinity in rubrene films deposited on IL mixtures. Figure 11b further illustrates that ILs deposited on Au promote greater rubrene crystallinity compared to those on ITO. Additionally, the use of IL mixtures significantly enhances rubrene crystallization, resulting in larger crystallite sizes than those observed in films prepared with pure ILs. Viscosity likely plays a crucial role in governing molecular mobility and nucleation kinetics. The longer-chain IL ([C_8_C_1_im]­[OTf]) exhibits higher viscosity (see Table S1 of Supporting Information), which may slow molecular diffusion and stabilize intermediate nucleation states, leading to larger and more ordered rubrene crystals. In contrast, the lower viscosity of the short-chain IL ([C_2_C_1_im]­[OTf]) could facilitate faster molecular rearrangement, enhancing diffusion at the IL–rubrene interface. The improved crystallinity observed with the IL mixture suggests that it balances these effects, enabling sufficient molecular mobility for optimal rubrene diffusion while maintaining the structural ordering benefits of the longer-chain IL.

These findings underscore the critical role of the substrate and the potential of using IL mixture films in polymorphism control and optimization of the crystalline quality of rubrene films. Overall, the present results provide valuable insights for future applications of rubrene in organic electronic devices.

## Conclusions

This study provided a comprehensive analysis of thin films composed of mixtures of [C_2_C_1_im]­[OTf] and [C_8_C_1_im]­[OTf], which were codeposited via vacuum thermal evaporation onto ITO, Ag, and Au substrates. The results demonstrated how varying the ratio of each IL in the mixture affected droplet formation, surface coverage, and overall film structure.

Pure [C_2_C_1_im]­[OTf] formed smaller, more spherical droplets, while pure [C_8_C_1_im]­[OTf] resulted in larger, more irregular droplets. SEM revealed that increasing the proportion of [C_8_C_1_im]­[OTf] in the mixture film led to larger droplet sizes, lower contact angles, and consequent better wettability. Coalesced films were observed on Au surfaces when the amount of [C_8_C_1_im]­[OTf] was larger than that of [C_2_C_1_im]­[OTf]. Detailed examination of the IL film morphology near metal-ITO interfaces highlighted increased mobility and droplet coalescence at the interfaces, suggesting that IL pairs migrated toward the metallic interfaces and influenced the film structure. XPS confirmed peak intensity variations corresponding to the mixtures’ different IL proportions. The IL-assisted vapor deposition of rubrene showed significant differences depending on the IL and the substrate type used. XRD demonstrated that deposition onto IL-coated surfaces improved crystallization. Notably, a mixture of [C_2_C_1_im]­[OTf] and [C_8_C_1_im]­[OTf] greatly enhanced rubrene crystallinity on both ITO and Au surfaces, indicating that the mixed IL environment provided an optimal balance for solubility and crystallization.

Overall, this study underscored the critical role of IL mixture composition and substrate type in determining the thin film morphology and further highlighted the potential of IL film mixtures for enhancing the crystallization of organic compounds. The findings suggested that carefully tailoring the IL environment and substrate interactions could improve the properties and quality of deposited films, with significant implications for various applications in materials science and electronics.

## Supplementary Material



## Notes

Details on the vapor deposition methodology, along with optical images and SEM micrographs of IL films deposited on various solid substrates; XPS characterization data for both pure IL films and IL mixture films; experimental results on the morphology, structure, and optical spectra of rubrene films obtained via IL-assisted vapor deposition (PDF)
