# Supplementary material for: Tailoring Morphology and Wetting Behavior of Films of Ionic Liquid Mixtures
Source: Langmuir. 2025 Mar 27;41(13):9086–99. doi: 10.1021/acs.langmuir.5c00653 (PMC12818764; doi:10.1021/acs.langmuir.5c00653)
Supplement: Supplementary file 1 [file la5c00653_si_001.pdf]

Supporting Information (SI)

Tailoring Morphology and Wetting Behavior of Films of Ionic Liquid Mixtures

Soraia R. M. R. Silva,[†] *Rita M. Carvalho,*[†] *Oleksandr Bondarchuk,*[‡, ‡‡, ‡‡‡] *Gonçalo N. P. Oliveira,*[#] *João P. Araújo,*[#] *Margarida Bastos,*[†] *Luís M. N. B. F. Santos,*[†] *and José C. S. Costa*[*,†]

*Corresponding author

Email: jose.costa@fc.up.pt

[†] CIQUP/Institute of Molecular Sciences (IMS), Departamento de Química e Bioquímica, Faculdade de Ciências, Universidade do Porto, Rua do Campo Alegre s/n, 4169-007 Porto, Portugal.

[‡] International Iberian Nanotechnology Laboratory, Av. Mestre José Veiga, s/n, 4715-330, Braga, Portugal.

[‡‡] SPIN-Lab Centre for Microscopic Research on Matter, University of Silesia in Katowice, 75 Pułku Piechoty Str. 1A, Chorzów 41-500, Poland.

[‡‡‡] Institute of Chemistry, University of Silesia in Katowice, 9 Szkolna Str., 40-006 Katowice, Poland.

[#] IFIMUP, Instituto de Física de Materiais Avançados, Nanotecnologia e Fotónica, Departamento de Física e Astronomia, Faculdade de Ciências, Universidade do Porto, Rua do Campo Alegre s/n, 4169-007 Porto, Portugal.

Details on the vapor deposition methodology, along with optical images and SEM micrographs of IL films deposited on various solid substrates, are provided. Additionally, XPS characterization data for both pure IL films and IL mixture films are presented. The Supporting Information also includes experimental results on the morphology, structure, and optical spectra of rubrene films obtained via IL-assisted vapor deposition.

Index

1. **1.** Relevant Properties of the ILs Studied P. S6
2. **2.** Physical Vapor Deposition P. S6
3. **3.** Substrates P. S8
4. **4.** Thin Film Morphology P. S9
5. **5.** X-Ray Photoelectron Spectroscopy (XPS) P. S15
6. **6.** UV-Vis Spectroscopy P. S27
7. **7.** X-Ray Diffraction P. S28
8. **8.** Experimental Data for the Deposition of ILs P. S30
9. **9.** References P. S31

List of Tables

Table S1. CAS registry number, molar mass, density, viscosity, melting temperature, and superficial tension values for the ionic liquids [C_2C_1im][OTf] and [C_8C_1im][OTf]. P. S6

Table S2. Experimental C_{cation} : C_{anion}, N_{cation} : $F_{anion,}$ and N_{cation} : S_{anion} ratios derived from the XPS data. P. S26

Table S3. Experimental data for the deposition of IL mixtures with varying proportions of each IL and different film thicknesses. P. S30

List of Figures

Figure S1. Schematic representation of the vacuum thermal evaporation methodology and schematic detail of the PVD of ionic liquids by thermal evaporation from a Knudsen cell. P. S6

Figure S2. Pictures of the exterior and interior parts of the ThinFilmVD system. P. S6

Figure S3. Schematic representation of the ovens/evaporation sources of the ThinFilmVD apparatus and image of an individual oven. P. S7

Figure S4. Schematic representation and images of the substrate support system. P. S7

Figure S5. Schemes and pictures of the substrates used in this work. P. S8

Figure S6. Morphology of the substrates. P. S8

Figure S7. Schematic illustration of the typical mechanisms of nucleation and growth of ionic liquid films obtained by vapor deposition. P. S9

Figure S8. Morphology of thin films (100 ML) composed of mixtures of [C_2C_1im][OTf] and [C_8C_1im][OTf], deposited on Au/ITO/glass surfaces by simultaneous deposition of both ILs, with varying individual flow rates for each IL. P. S10

Figure S9. Morphology of thin films (50 ML) composed of mixtures of [C_2C_1im][OTf] and [C_8C_1im][OTf], deposited on Au/ITO/glass surfaces by simultaneous deposition of both ILs, with varying individual flow rates for each IL. P. S10

Figure S10. Morphology of thin films comprising mixtures of [C_2C_1im][OTf] and [C_8C_1im][OTf], deposited on ITO/glass by simultaneous deposition of both ILs. Images characterized at different time intervals. Histograms displaying the droplet size distribution. P. S11

Figure S11. Morphology of thin films comprising mixtures of [C_2C_1im][OTf] and [C_8C_1im][OTf], deposited on Au/ITO/glass by simultaneous deposition of both ILs. Images characterized at different time intervals. Histograms displaying the droplet size distribution. P. S12

Figure S12. Morphology of thin films of [C_2C_1im][OTf], [C_8C_1im][OTf], and mixtures of [C_2C_1im][OTf] and [C_8C_1im][OTf] deposited simultaneously on ITO and Au. P. S13

Figure S13. Morphology of rubrene films deposited on ITO-coated glass and Au/ITO-coated glass surfaces. P. S14

Figure S14. XPS survey spectra of the ITO/glass surface (substrate exposed to air). P. S15

Figure S15. XPS survey spectra of the ITO/glass surface after the removal of adventitious carbon. P. S15

Figure S16. XPS survey spectra of the Ag/ITO/glass surface (substrate exposed to air). P. S16

Figure S17. XPS survey spectra of the Ag/ITO/glass surface after the removal of adventitious carbon. P. S16

Figure S18. XPS survey spectra of the Au/ITO/glass surface. P. S17

Figure S19. XPS survey spectrum of [C_2C_1im][OTf] deposited on ITO/glass. P. S18

Figure S20. XPS survey spectrum of [C_2C_1im][OTf] deposited on Au/ITO/glass. P. S18

Figure S21. XPS survey spectrum of [C_8C_1im][OTf] deposited on ITO/glass. P. S19

Figure S22. XPS survey spectrum of [C_8C_1im][OTf] deposited on Au/ITO/glass. P. S19

Figure S23. XPS survey spectrum of a mixture of [C_2C_1im][OTf] and [C_8C_1im][OTf] (x{[C_8C_1im][OTf]} = 0.1), deposited on ITO/glass surface. P. S20

Figure S24. XPS survey spectrum of a mixture of [C_2C_1im][OTf] and [C_8C_1im][OTf] (x{[C_8C_1im][OTf]} = 0.1), deposited on Au/ITO/glass surface. P. S20

Figure S25. XPS survey spectrum of a mixture of [C_2C_1im][OTf] and [C_8C_1im][OTf] (x{[C_8C_1im][OTf]} = 0.4), deposited on ITO/glass surface. P. S21

Figure S26. XPS survey spectrum of a mixture of [C_2C_1im][OTf] and [C_8C_1im][OTf] (x{[C_8C_1im][OTf]} = 0.4), deposited on Au/ITO/glass surface. P. S21

Figure S27. XPS survey spectrum of a mixture of [C_2C_1im][OTf] and [C_8C_1im][OTf] (x{[C_8C_1im][OTf]} = 0.8), deposited on ITO/glass surface. P. S22

Figure S28. XPS survey spectrum of a mixture of [C_2C_1im][OTf] and [C_8C_1im][OTf] (x{[C_8C_1im][OTf]} = 0.8), deposited on Au/ITO/glass surface. P. S22

Figure S29. High-resolution XPS spectra of IL films deposited on ITO/glass surfaces by simultaneous deposition of [C_2C_1im][OTf] and [C_8C_1im][OTf], with a varying mole fraction of each IL in the resulting film. The XPS spectra were acquired for C 1s, N 1s, F 1s, O 1s, and S 2p. P. S23

Figure S30. High-resolution XPS spectra of IL films deposited on Au/ITO/glass surfaces by simultaneous deposition of [C_2C_1im][OTf] and [C_8C_1im][OTf], with a varying mole fraction of each IL in the resulting film. The XPS spectra were acquired for C 1s, N 1s, F 1s, O 1s, and S 2p. P. S24

Figure S31. High-resolution XPS spectra of IL films deposited on ITO/glass surfaces by simultaneous deposition of [C_2C_1im][OTf] and [C_8C_1im][OTf], with a varying mole fraction of each IL in the resulting film. The XPS spectra were acquired for In 3d and Sn 3d. P. S25

Figure S32. High-resolution XPS spectra of IL films deposited on Au/ITO/glass surfaces by simultaneous deposition of [C_2C_1im][OTf] and [C_8C_1im][OTf], with a varying mole fraction of each IL in the resulting film. The XPS spectra were acquired for Au 4f. P. S25

Figure S33. UV-vis absorption spectra comparison of rubrene films deposited on various surfaces. P. S27

Figure S34. Literature data of the XRD pattern of rubrene single crystals. P. S28

Figure S35. X-ray diffraction patterns of rubrene films deposited on various surfaces. P. S28

1. Relevant Properties of the ILs Studied

Table S1. CAS registry number (CAS), molar mass (*M*), density (*ρ*), viscosity (*η*), melting temperature (T_m), and superficial tension (*γ*) values for the ionic liquids [C_2C_1im][OTf] and [C_8C_1im][OTf].

Ionic Liquid	CAS	*M* / g·mol^{-1}	*ρ* (298K) / g·cm^{-3}	*η* / mPa·s	T_m / K	*γ* / mN·m^{-1}
[C_2C_1im][OTf]	145022-44-2	260.23	1.39[1]	45.7[3] (298K)	258[5]	41.3[7] (298K)<br>39.2[8] (298K)
[C_8C_1im][OTf]	403842-84-2	344.39	1.19[2]	127.4[4] (297K)	253[6]	28.5[8] (298K)

2. Physical Vapor Deposition

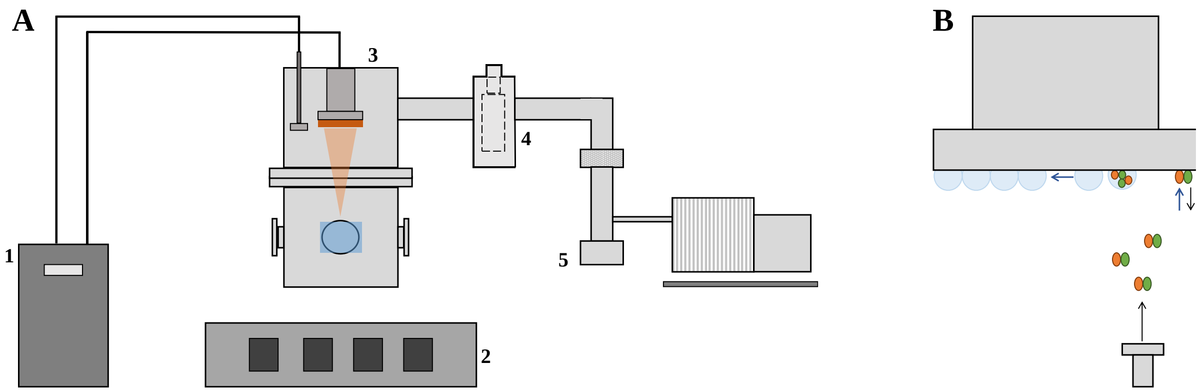

Figure S1. Schematic representation of the vacuum thermal evaporation methodology: (A) ThinFilmVD apparatus (1 – cooling system, 2 – instrumentation box, 3 – vacuum chamber, 4 – N_2 (l) metallic trap, 5 – vacuum pumping system); (B) schematic detail of the PVD of ionic liquids by thermal evaporation from a Knudsen cell.[9,10]

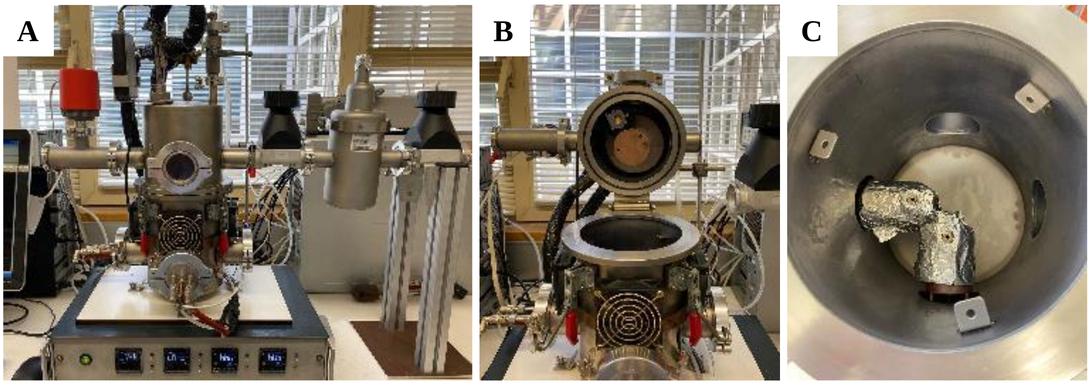

Figure S2. Pictures of the exterior (A) and interior parts (B and C) of the ThinFilmVD system. A dual-source thermal evaporation process was used to fabricate IL mixture films. For this purpose, two furnaces, each with independent temperature control, were employed, with each furnace housing a Knudsen effusion cell. A quartz crystal microbalance (QCM) is positioned near the substrates to enable real-time deposition rate monitoring.

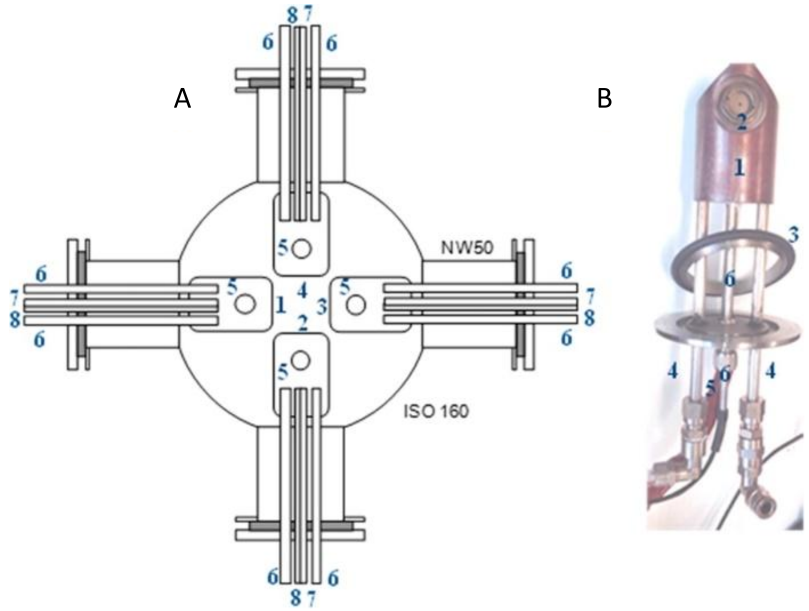

Figure S3. A – Schematic representation of the ovens/evaporation sources of the ThinFilmVD apparatus: 1, 2, 3, and 4 – individual copper ovens; 5 – cavity of the Knudsen cell screwing; 6 – air cooling tube; 7 – heater; 8, – Pt100 sensor. B – Image of an individual oven (top view): 1 – copper block; 2 – Knudsen cell; 3 – Viton O-ring; 4 – cooling system; 5 – heater; 6 – Pt100.[9]

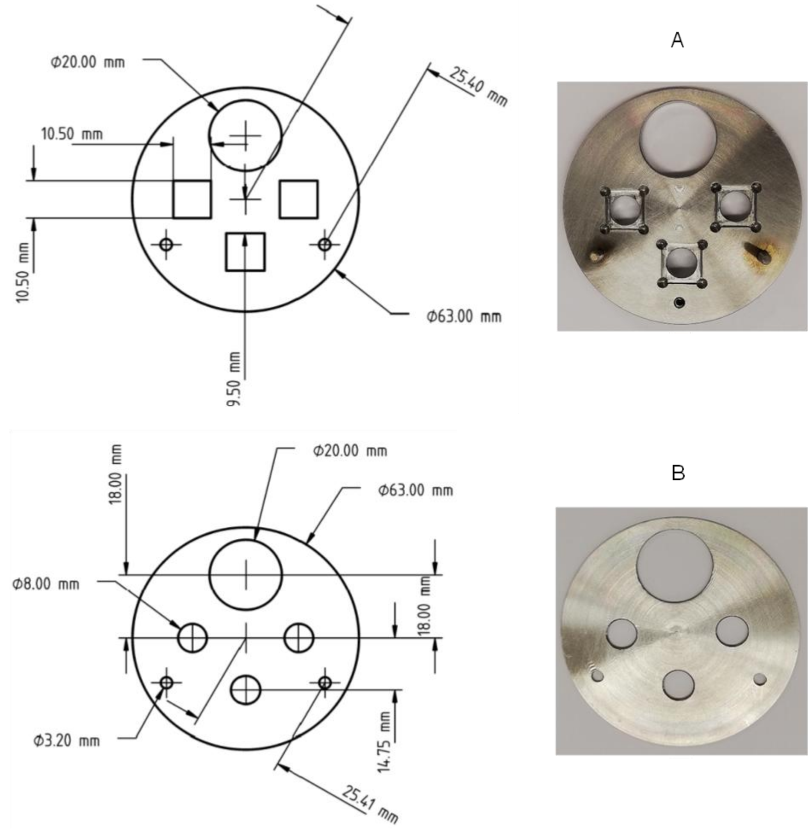

Figure S4. Schematic representation (left) and images (right) of the substrate support system: A – support for the ITO/glass substrates (up to 3 samples); B – disk used to fix the substrates, also serving as a shadow mask.

3. Substrates

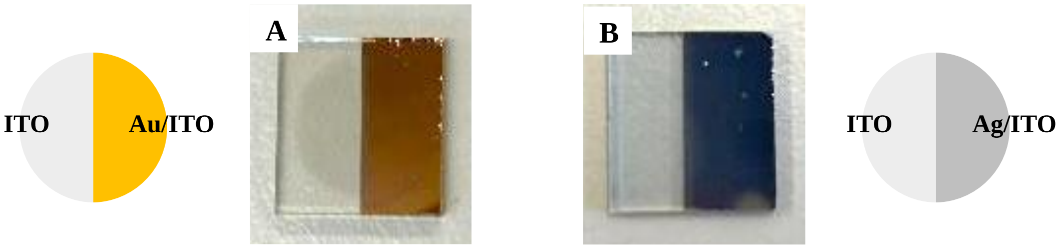

Figure S5. Schemes and pictures of the substrates used in this work – A) Au/ITO; B) Ag/ITO.

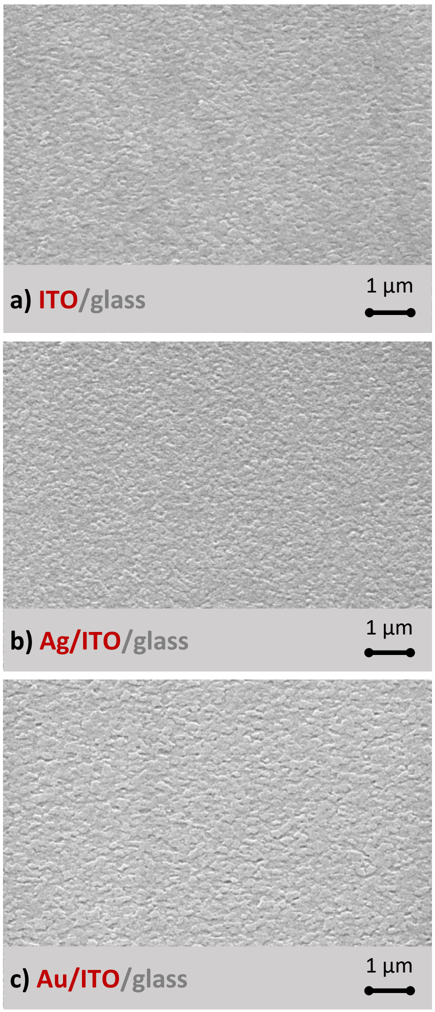

Figure S6. Morphology of the substrates: indium tin oxide (ITO)/glass surface (a); silver(Ag)/ITO/glass surface (b); gold(Au)/ITO/glass surface (c). Micrographs were acquired through high-resolution scanning electron microscopy (SEM) using a secondary electron detector (SED). Lateral views at 45° were obtained with a magnification of 25000×. The ITO/glass substrates, measuring 10 mm × 10 mm × 1.1 mm, were commercially obtained from Praezisions Glas & Optik GmbH. The ITO films have an approximate thickness of 180 nm. The ITO substrates underwent a sputtering process to obtain the metal surfaces (Ag/ITO and Au/ITO), coating the ITO surfaces with a metal film of 100 nm thickness.

4. Thin Film Morphology

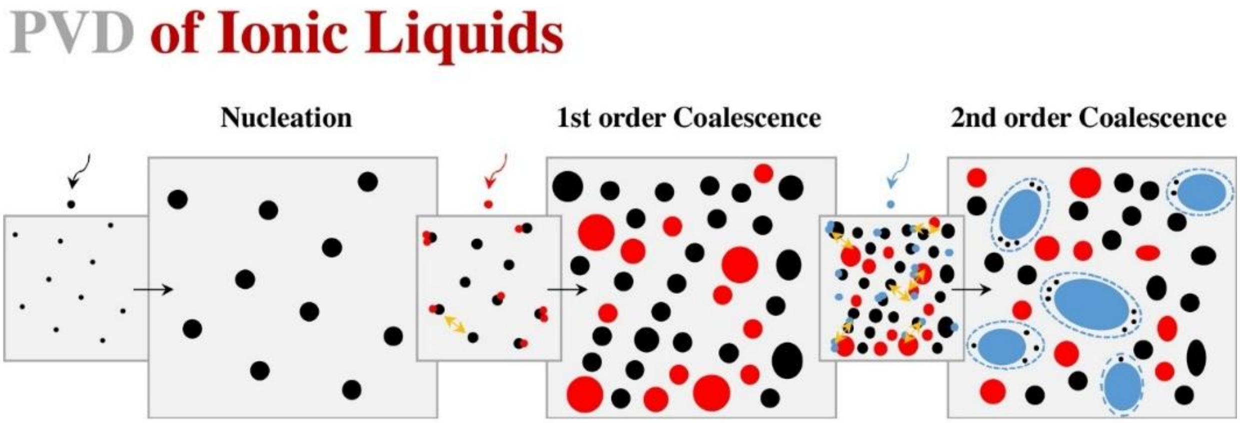

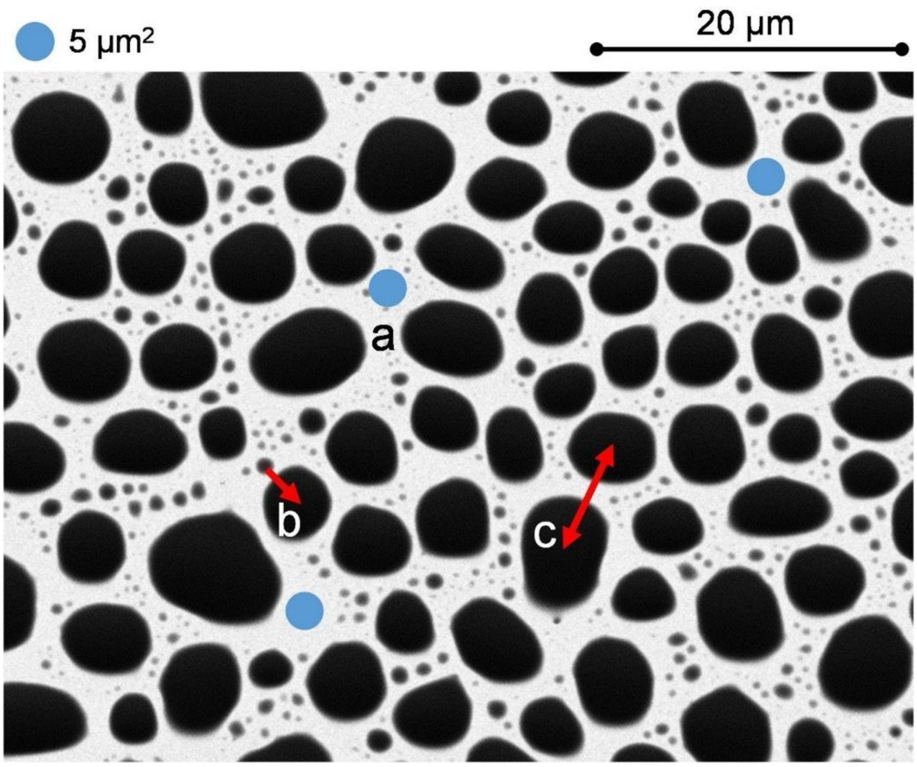

Figure S7. Schematic illustration of the typical mechanisms of nucleation and growth of ionic liquid films obtained by vapor deposition: minimum free area to promote nucleation (MFAN); first-order coalescence; second-order coalescence.[10] SEM image of IL deposited on ITO surface showing the three main processes of nucleation and growth of ILs droplets fabricated by physical vapor deposition: minimum free area to promote nucleation (a); first order coalescence (b); second order coalescence (c).

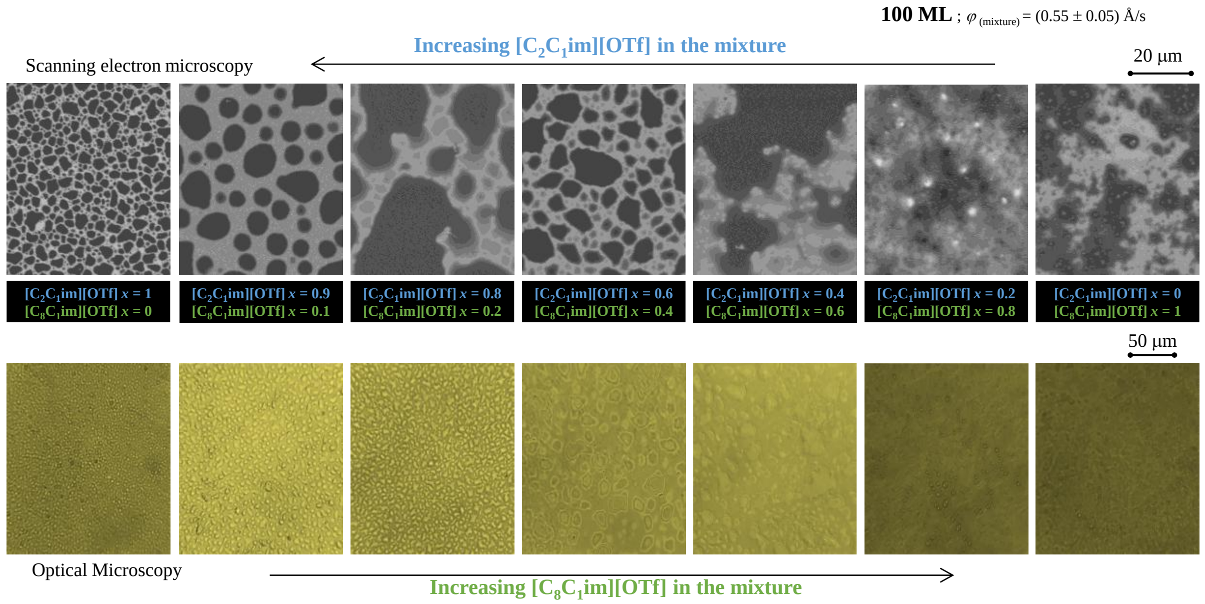

Figure S8. Morphology of thin films composed of mixtures of [C_2C_1im][OTf] and [C_8C_1im][OTf], deposited on Au/ITO/glass surfaces by simultaneous deposition of both ILs, with a varying mole fraction (x) of each IL in the resulting film. Top-view images were acquired by SEM using a backscattered electron detector, as well as through optical microscopy. These films theoretically consist of 100 monolayers (ML).

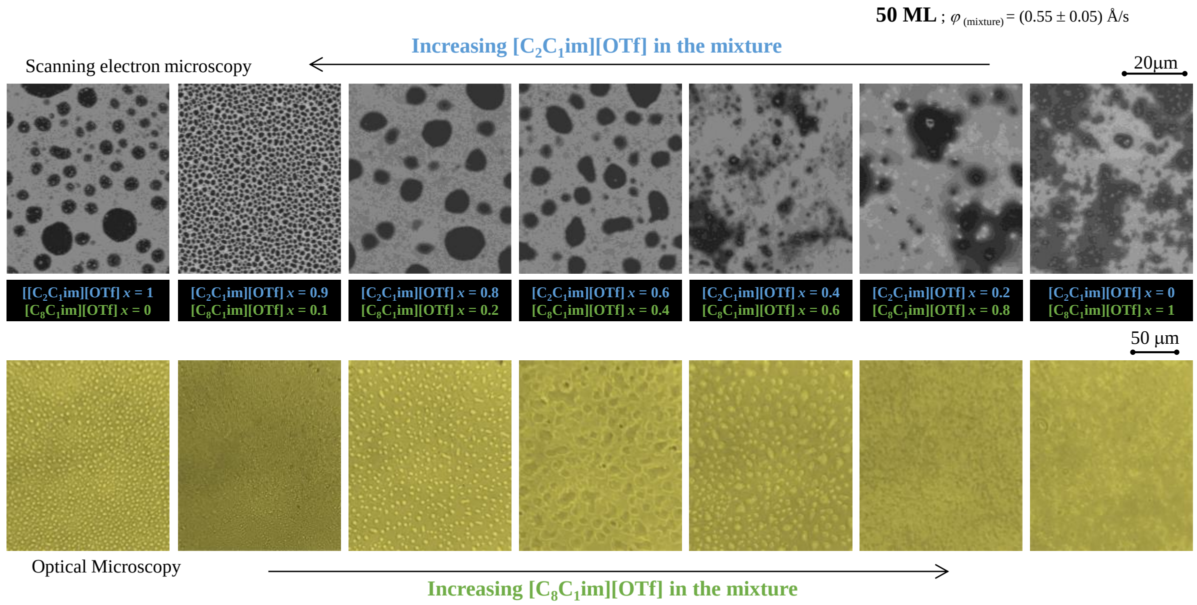

Figure S9. Morphology of thin films composed of mixtures of [C_2C_1im][OTf] and [C_8C_1im][OTf], deposited on Au/ITO/glass surfaces by simultaneous deposition of both ILs, with a varying mole fraction (x) of each IL in the resulting film. Top-view images were acquired by SEM using a backscattered electron detector, as well as through optical microscopy. These films theoretically consist of 50 monolayers (ML).

Mixture of [C_2C_1im][OTf] and [C_8C_1im][OTf]

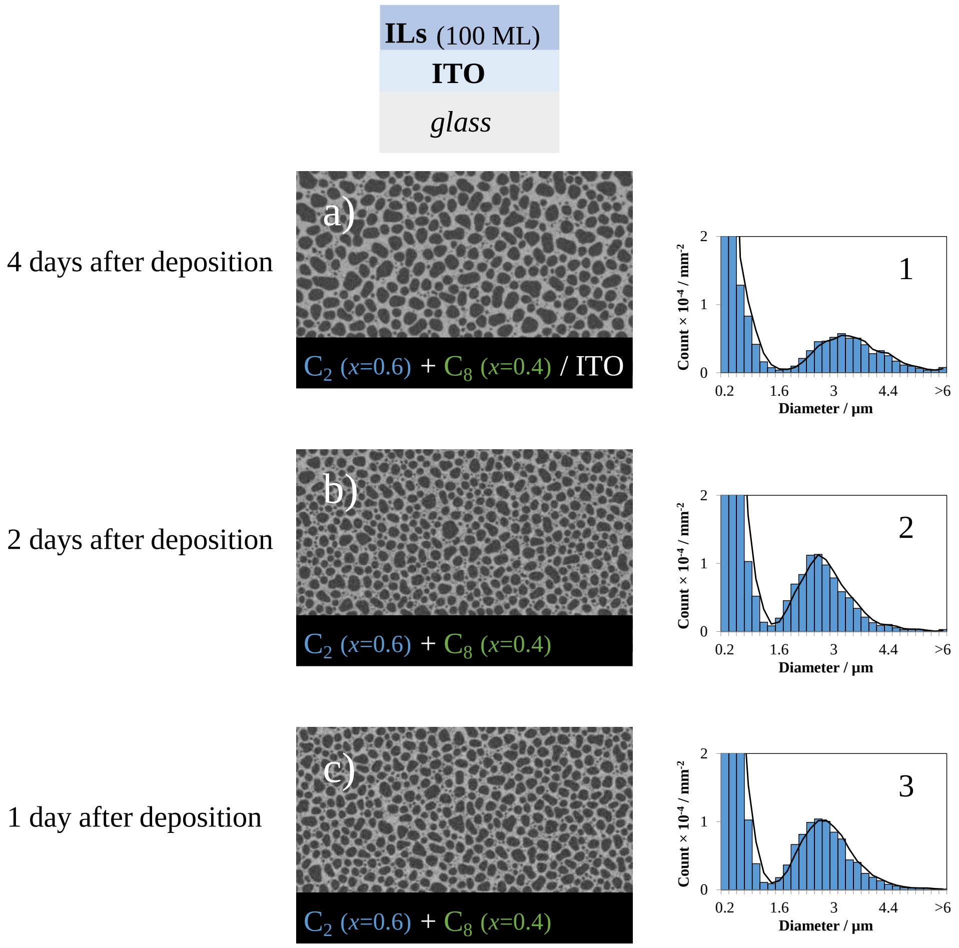

Figure S10. Morphology of thin films comprising mixtures (x represents the mole fraction of each IL in the mixture) of [C_2C_1im][OTf] (x=0.6) and [C_8C_1im][OTf] (x=0.4), deposited on ITO/glass surfaces by simultaneous deposition of both ILs (total deposition rate of 0.6 Å/s). These images depict IL film mixtures deposited under the same experimental conditions but characterized at different time intervals: 4 days after deposition (image a), 2 days after deposition (images b), and 1 day after deposition (image c). Top views were obtained using SEM with a backscattered electron detector. These films theoretically consist of 100 ML. Histograms 1-3 display the droplet size distribution for each sample.

Mixture of $[C_2C_1im][OTf]$ and $[C_8C_1im][OTf]$

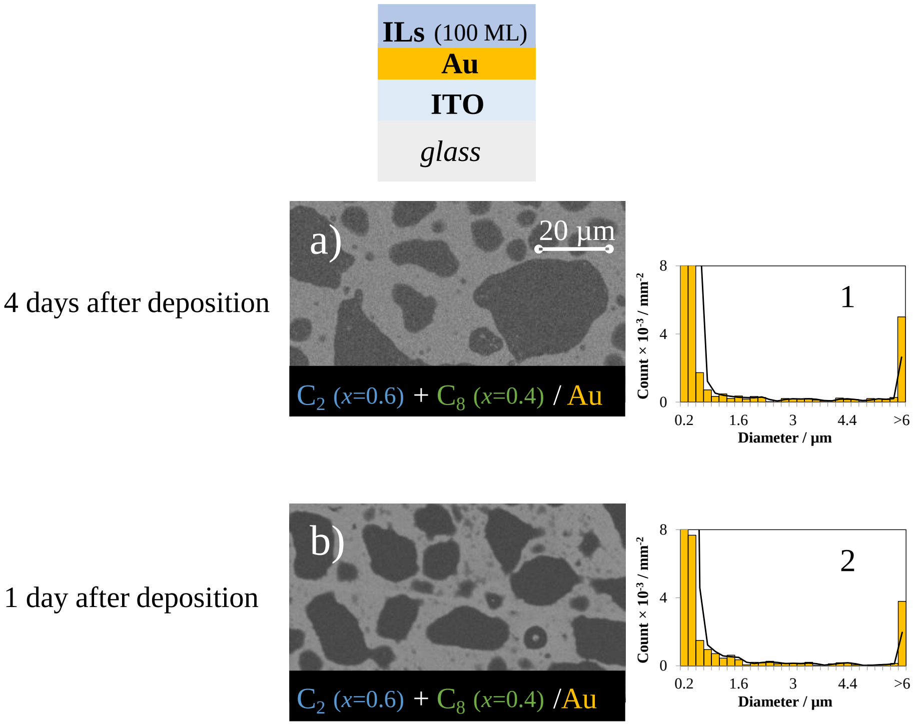

Figure S11. Morphology of thin films comprising mixtures (x represents the mole fraction of each IL in the mixture) of $[C_2C_1im][OTf]$ (x=0.6) and $[C_8C_1im][OTf]$ (x=0.4), deposited on Au/ITO/glass surfaces by simultaneous deposition of both ILs (total deposition rate of 0.6 Å/s). These images depict IL film mixtures deposited under the same experimental conditions but characterized at different time intervals: 4 days after deposition (image a), and 1 day after deposition (image b). Top views were obtained using SEM with a backscattered electron detector. These films theoretically consist of 100 ML. Histograms 1 and 2 display the droplet size distribution for each sample.

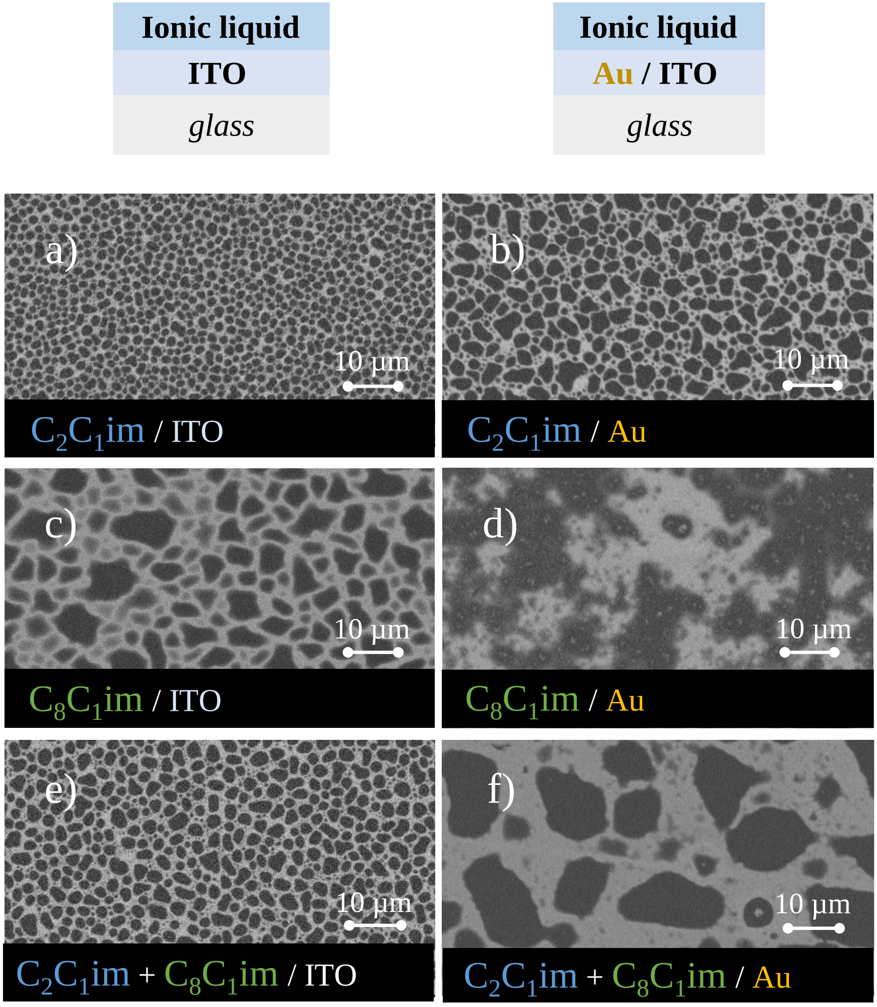

Figure S12. Morphology of thin films of [C_2C_1im][OTf] (a and b), [C_8C_1im][OTf] (c and d), and mixtures (e and f) of [C_2C_1im][OTf] (x=0.6) and [C_8C_1im][OTf] (x=0.4) (x represents the mole fraction of each IL in the mixture) deposited simultaneously on ITO (left images) and Au (right images). Top views were obtained using SEM with a backscattered electron detector. These films theoretically consist of 100 ML.

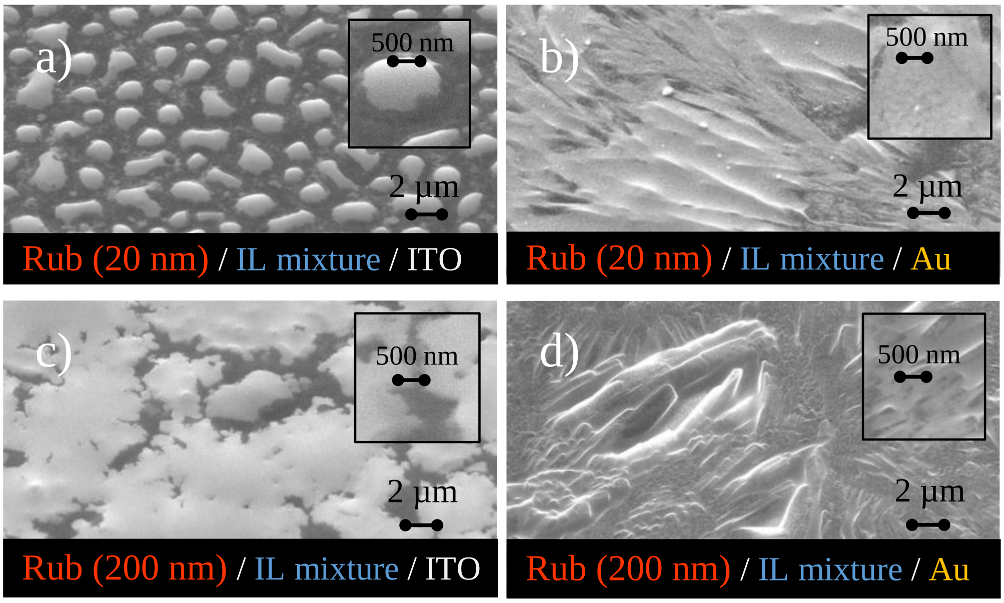

Figure S13. Morphology of rubrene (Rub) films deposited on ITO-coated glass (a and c) and Au/ITO-coated glass surfaces (b and d). Morphology of rubrene films with 20 (a and b) and 200 nm (c and d) deposited on substrates coated with a mixture of [C_2C_1im][OTf] (x=0.6) and [C_8C_1im][OTf] (x=0.4). The rubrene films were deposited using identical experimental parameters (same deposition rate and substrate temperature for all experiments). Lateral views acquired using a secondary electron detector.

5. X-Ray Photoelectron Spectroscopy (XPS)

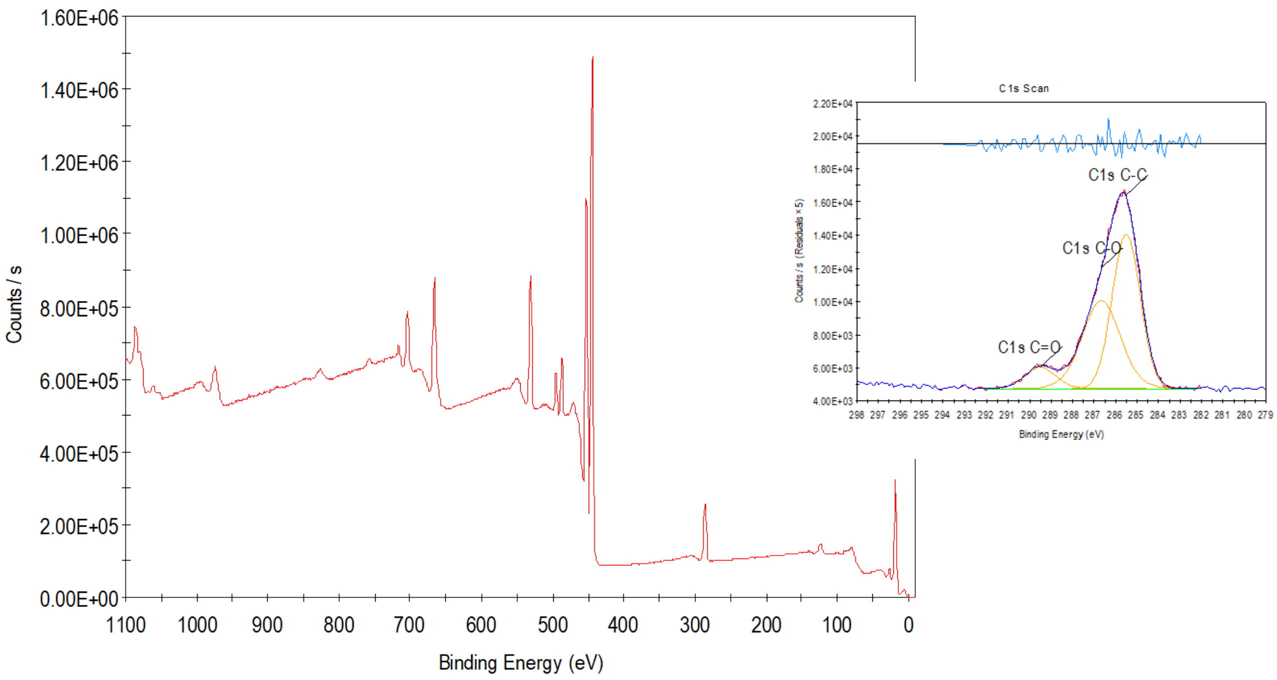

Figure S14. XPS survey spectra of the ITO/glass surface (substrate exposed to air). The C1s spectrum reveals adventitious carbon contamination on the surface, with detected C–C, C–O, and C=O components.

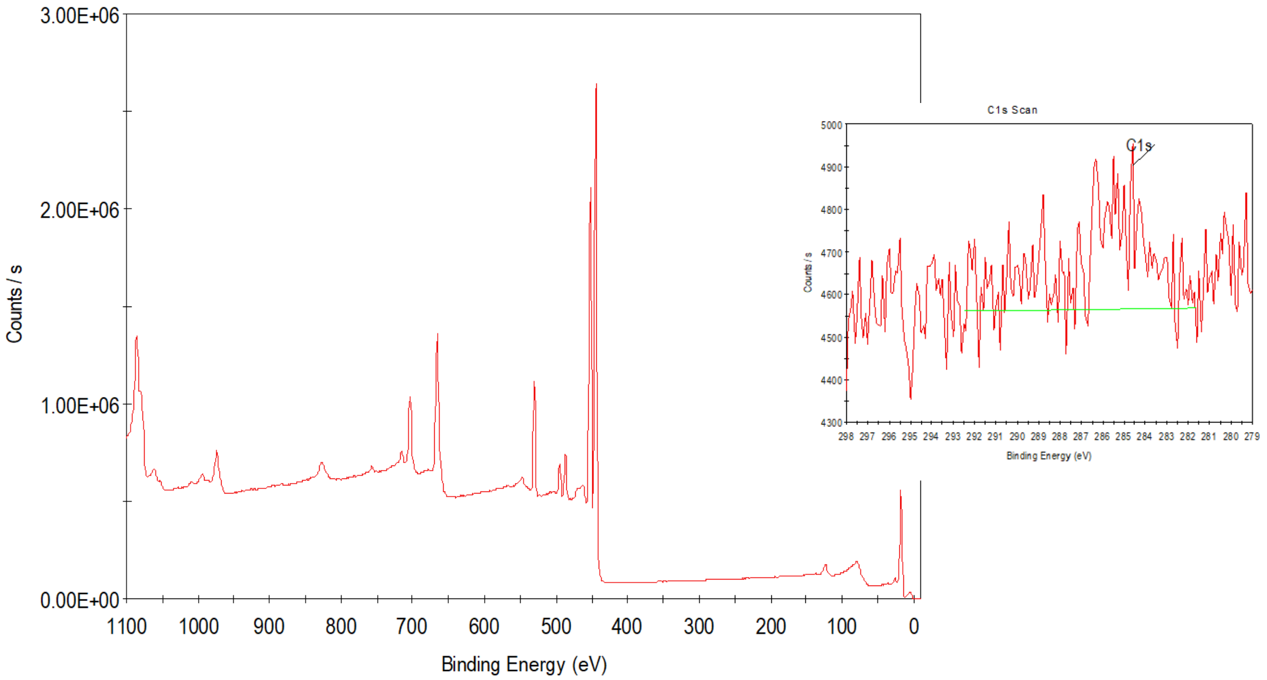

Figure S15. XPS survey spectra of the ITO/glass surface after the removal of adventitious carbon by argon sputtering.

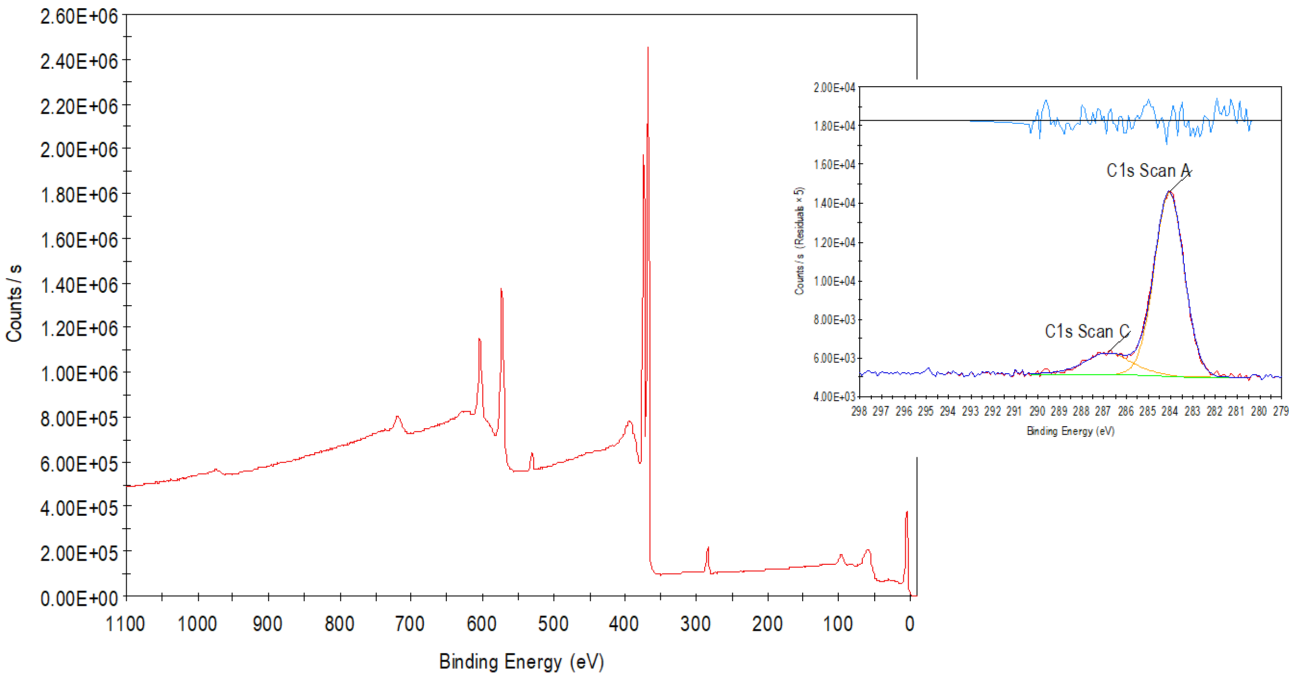

Figure S16. XPS survey spectra of the Ag/ITO/glass surface (substrate exposed to air). The C1s spectrum reveals adventitious carbon contamination on the surface.

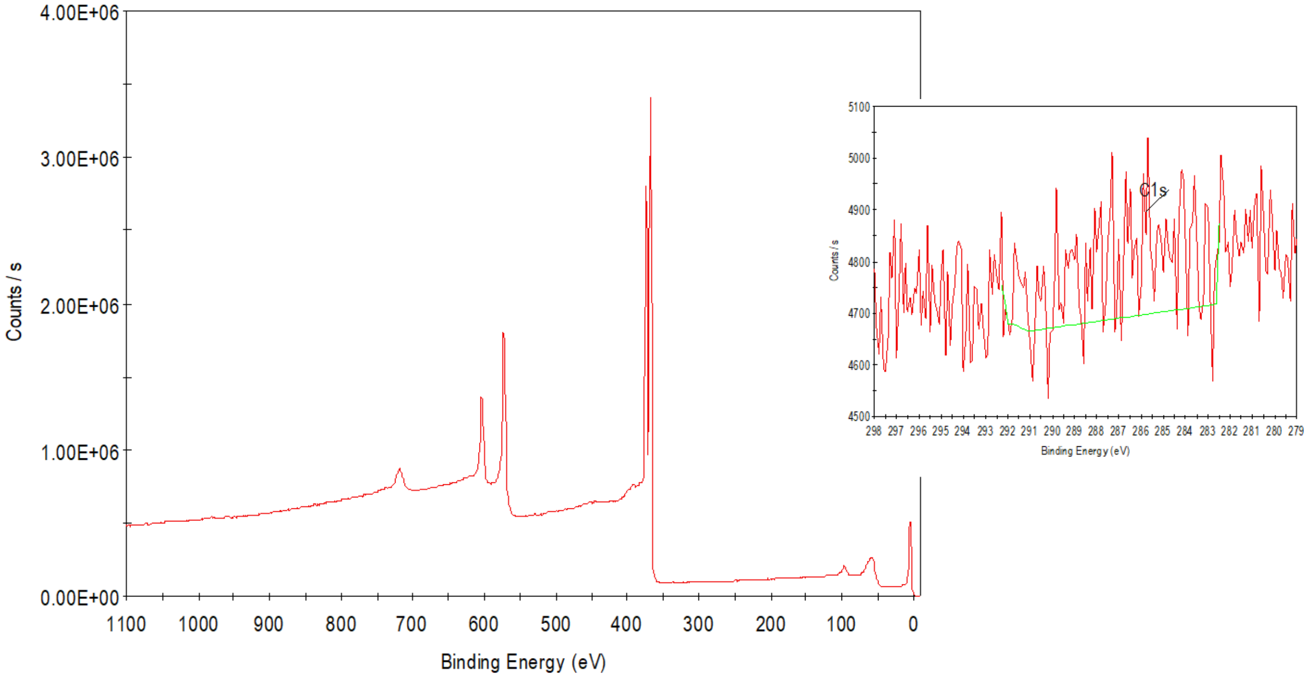

Figure S17. XPS survey spectra of the Ag/ITO/glass surface after the removal of adventitious carbon by argon sputtering.

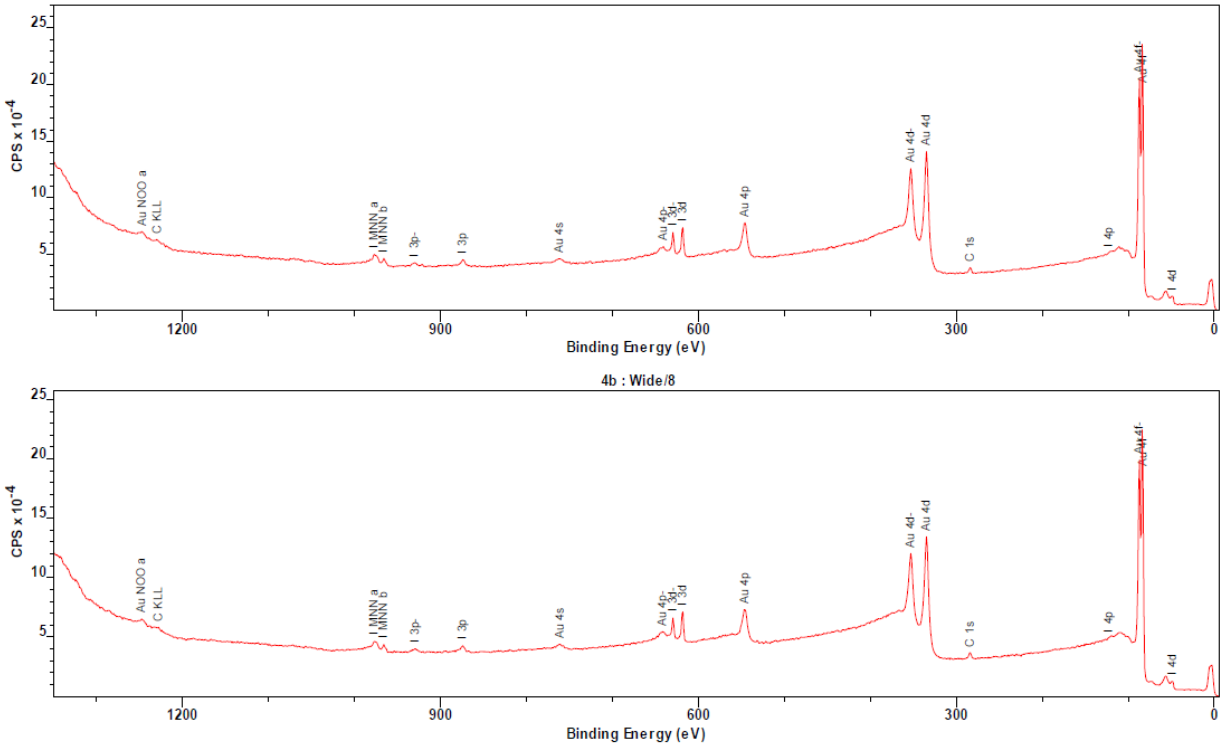

Figure S18. XPS survey spectra of the Au/ITO/glass surface. The two spectra correspond to data obtained from the analysis of two different areas, each measuring 300 µm × 700 µm.

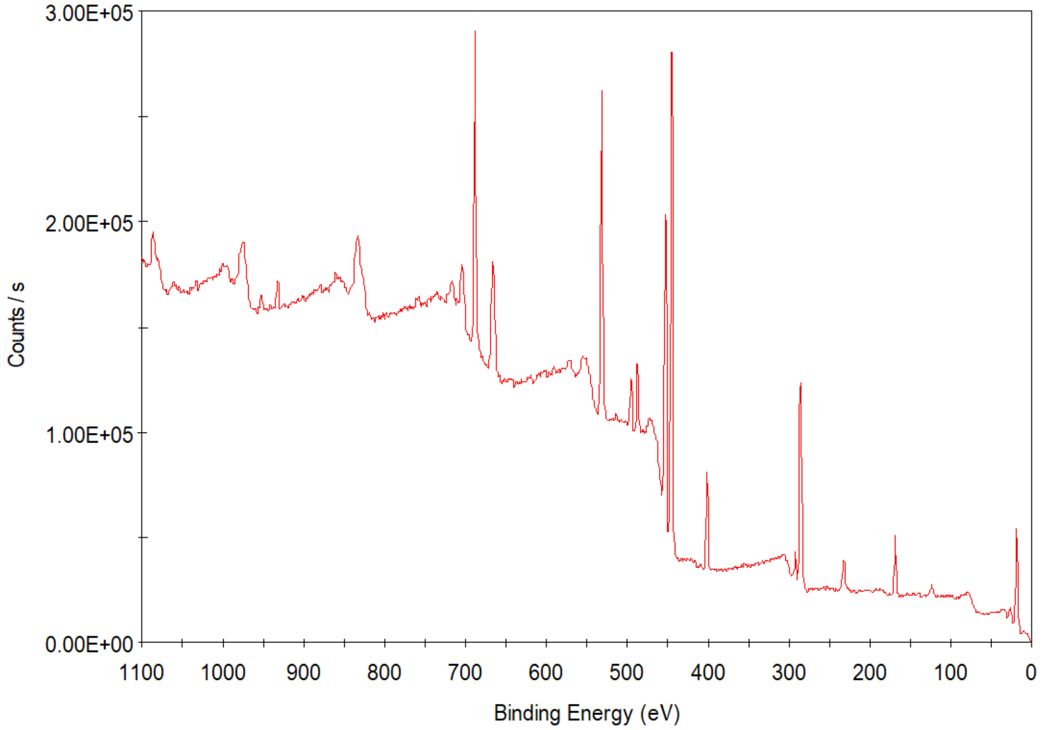

Figure S19. XPS survey spectrum of $[C_2C_1im][OTf]$ (100 ML) deposited on ITO/glass.

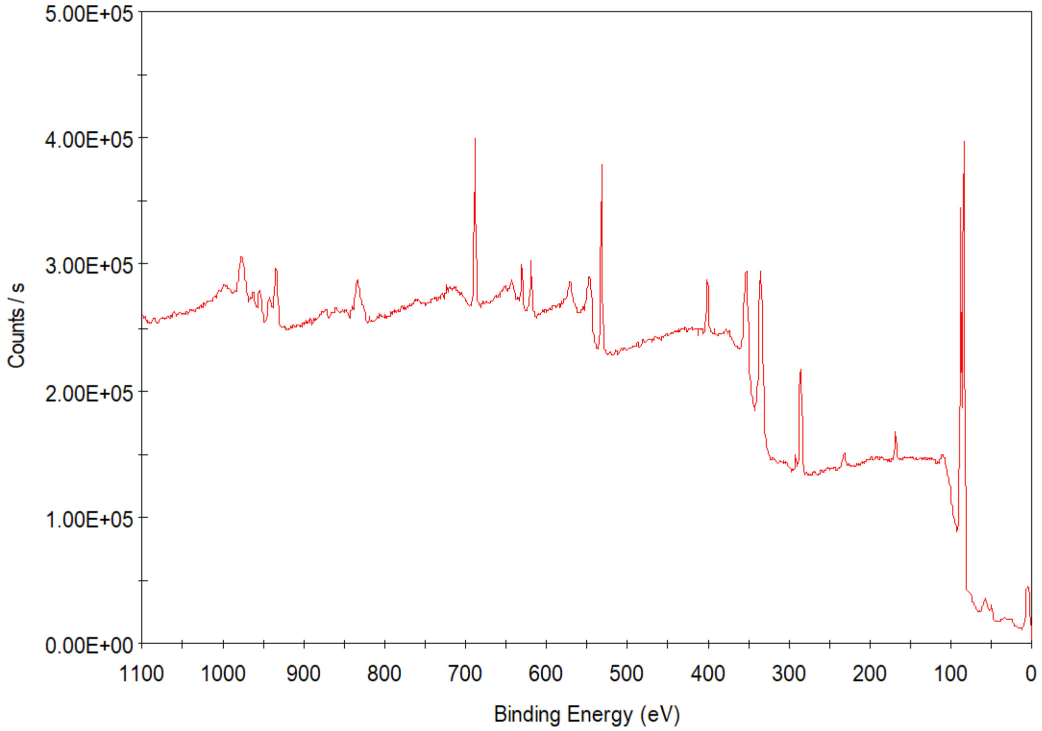

Figure S20. XPS survey spectrum of $[C_2C_1im][OTf]$ (100 ML) deposited on Au/ITO/glass.

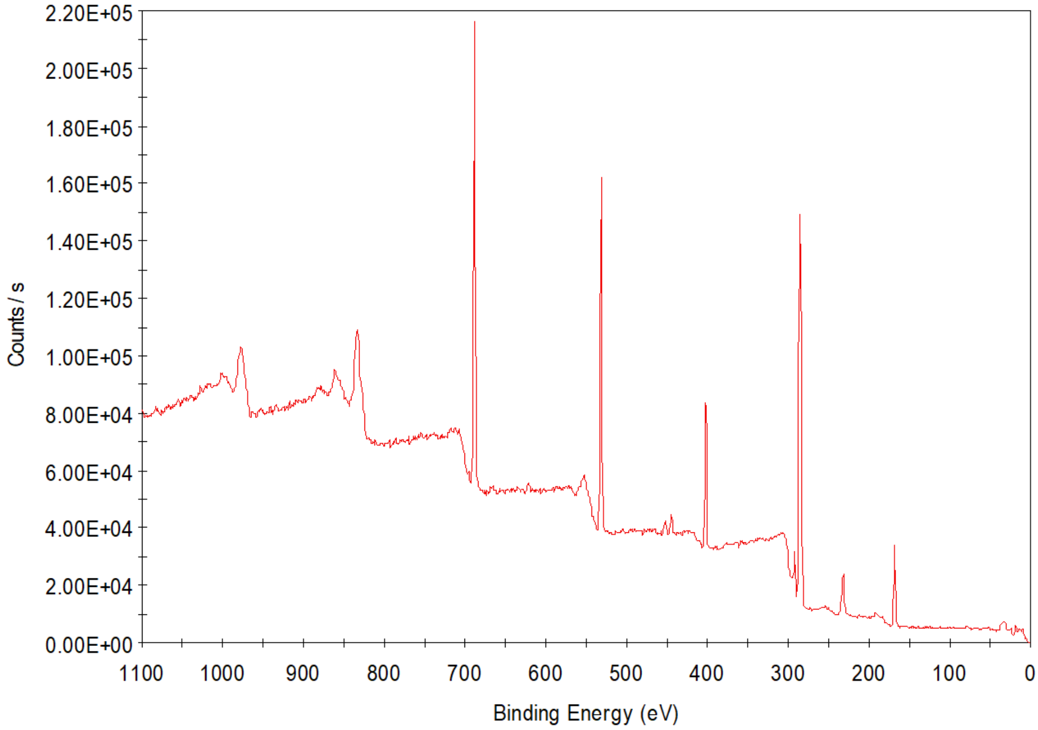

Figure S21. XPS survey spectrum of [C_8C_1im][OTf] (100 ML) deposited on ITO/glass.

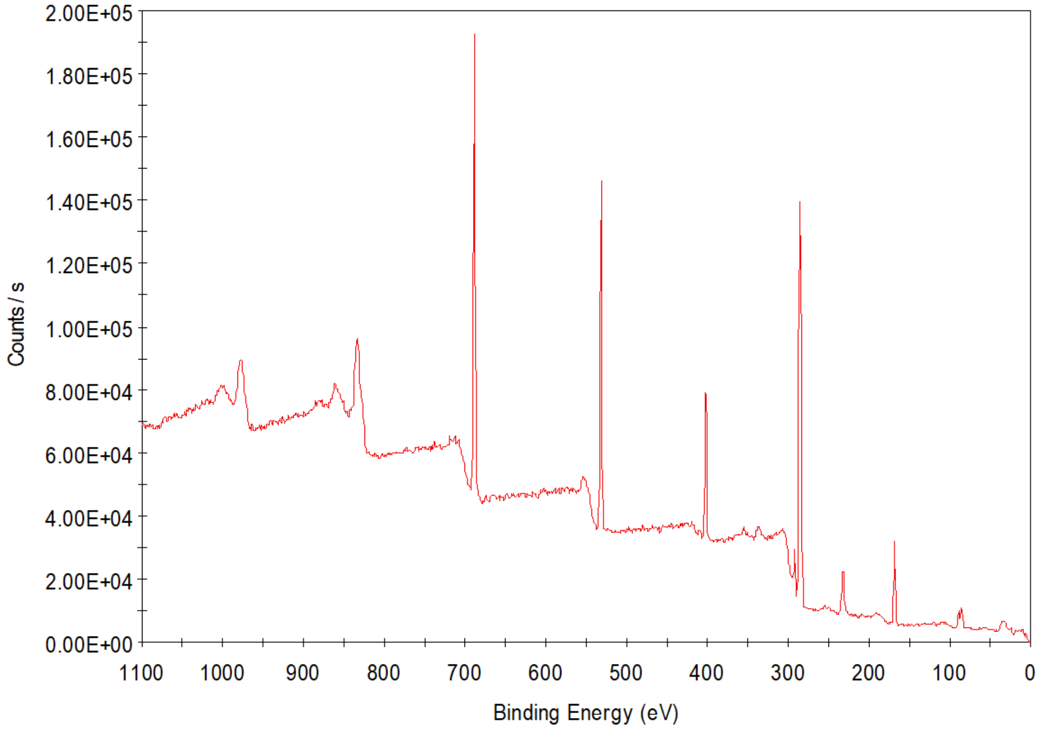

Figure S22. XPS survey spectrum of [C_8C_1im][OTf] (100 ML) deposited on Au/ITO/glass.

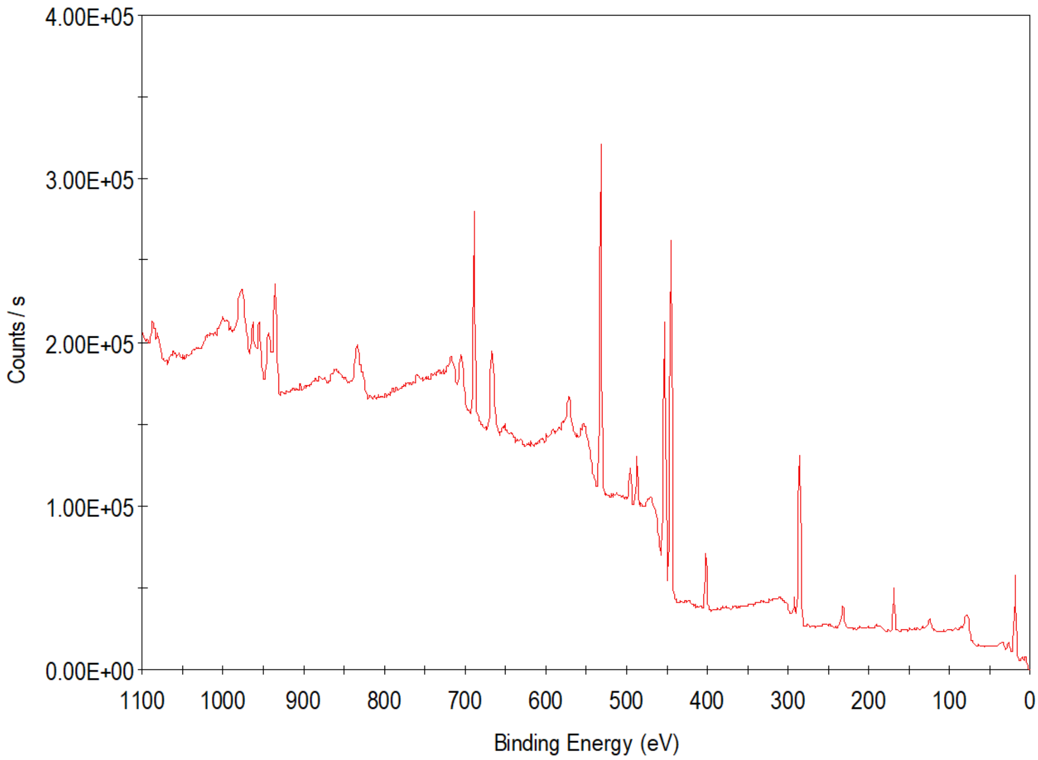

Figure S23. XPS survey spectrum of a mixture (100 ML) of [C_2C_1im][OTf] and [C_8C_1im][OTf] (mole fraction {[C_8C_1im][OTf]} = 0.1), deposited on ITO/glass surface.

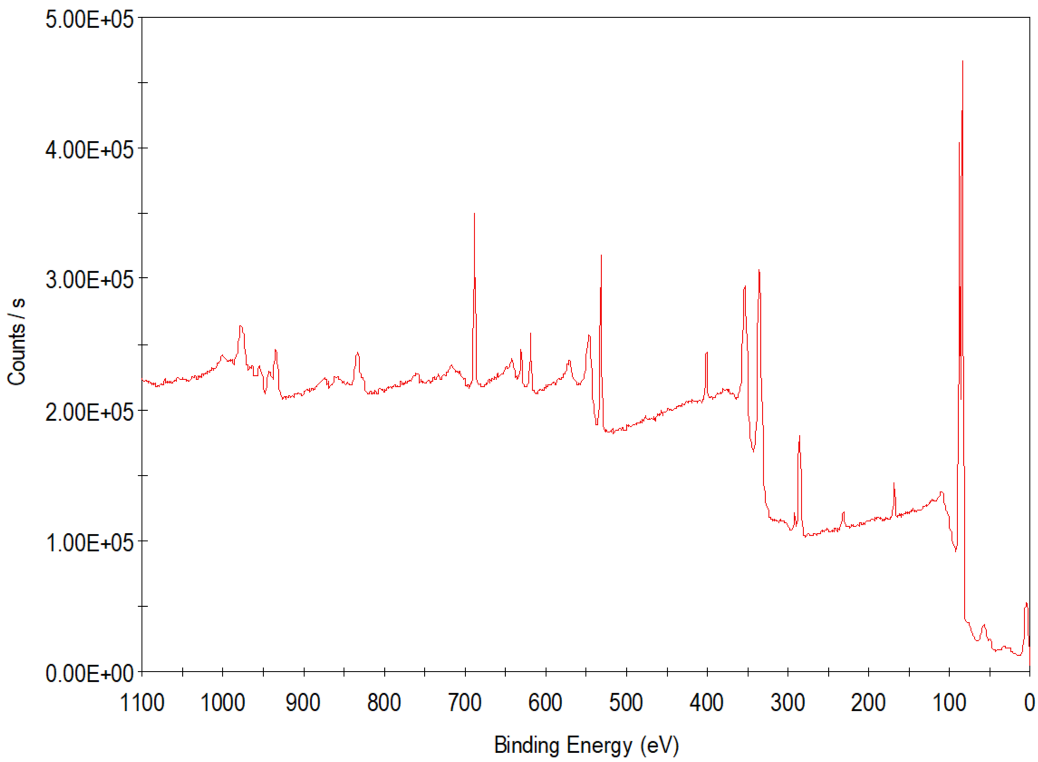

Figure S24. XPS survey spectrum of a mixture (100 ML) of [C_2C_1im][OTf] and [C_8C_1im][OTf] (mole fraction {[C_8C_1im][OTf]} = 0.1), deposited on Au/ITO/glass surface.

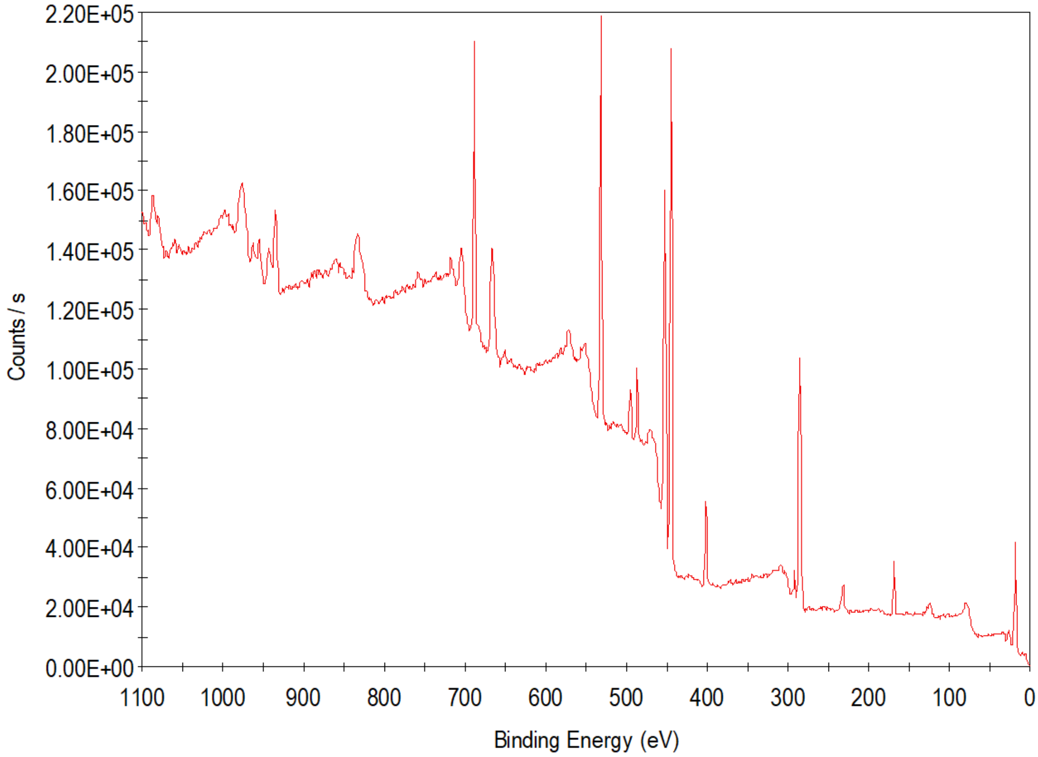

Figure S25. XPS survey spectrum of a mixture (100 ML) of [C_2C_1im][OTf] and [C_8C_1im][OTf] (mole fraction {[C_8C_1im][OTf]} = 0.4), deposited on ITO/glass surface.

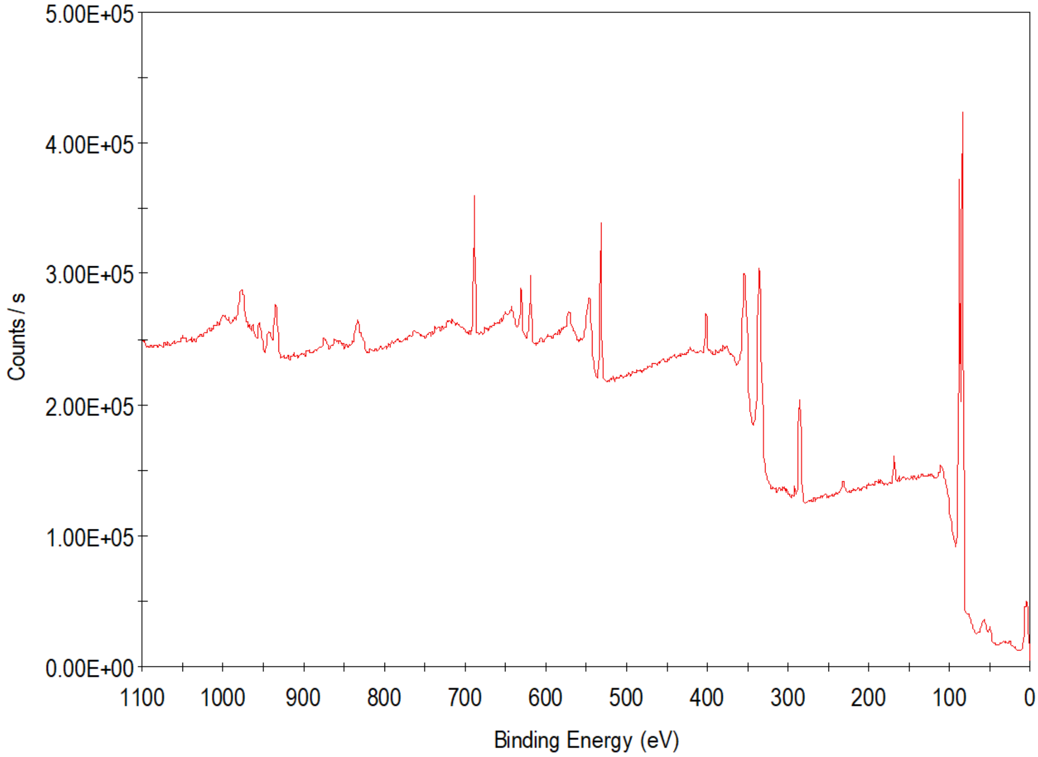

Figure S26. XPS survey spectrum of a mixture (100 ML) of [C_2C_1im][OTf] and [C_8C_1im][OTf] (mole fraction {[C_8C_1im][OTf]} = 0.4), deposited on Au/ITO/glass surface.

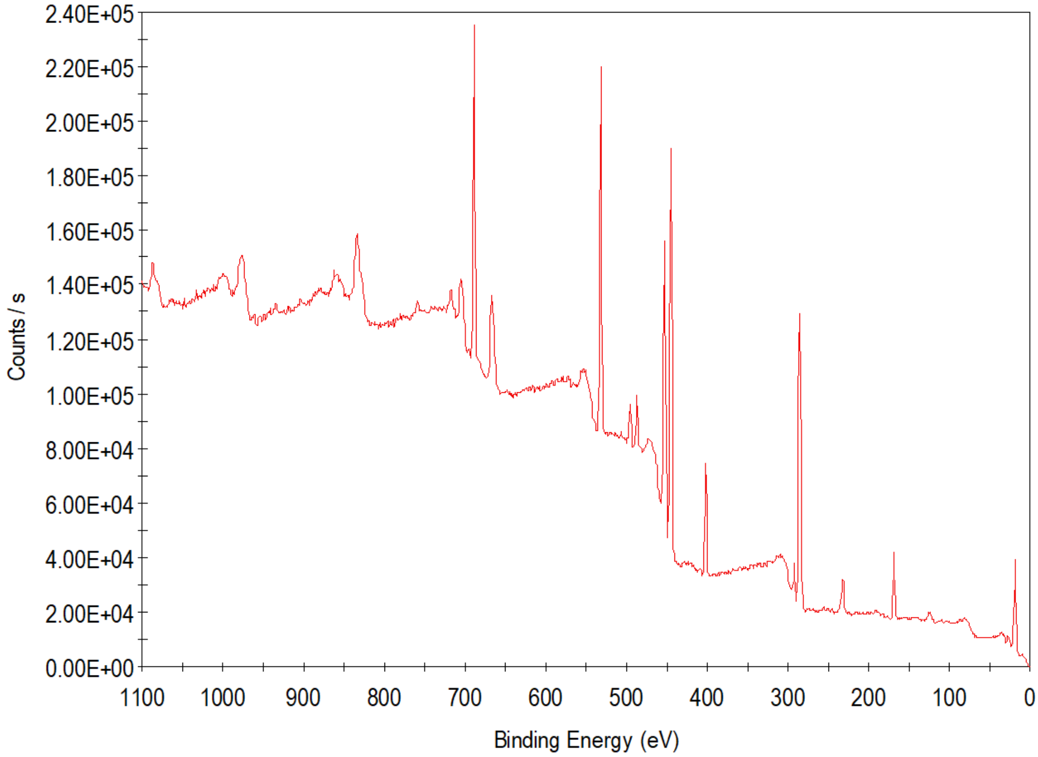

Figure S27. XPS survey spectrum of a mixture (100 ML) of $[C_2C_1im][OTf]$ and $[C_8C_1im][OTf]$ (mole fraction $\{[C_8C_1im][OTf]\} = 0.8$), deposited on ITO/glass surface.

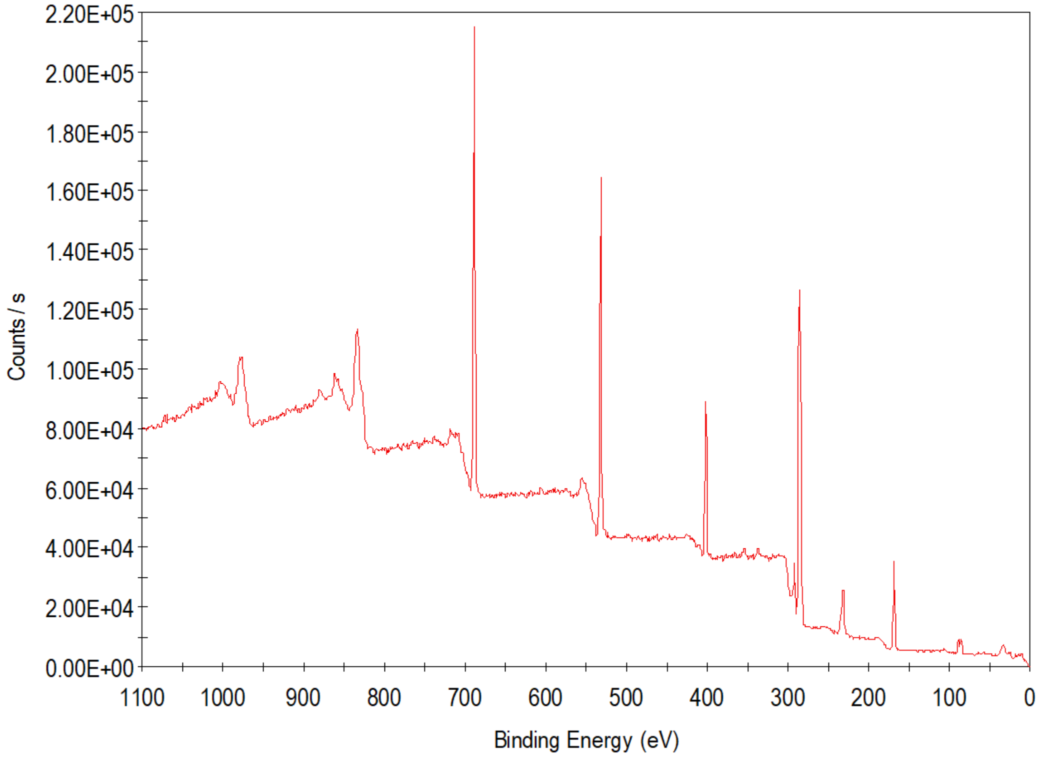

Figure S28. XPS survey spectrum of a mixture (100 ML) of $[C_2C_1im][OTf]$ and $[C_8C_1im][OTf]$ (mole fraction $\{[C_8C_1im][OTf]\} = 0.8$), deposited on Au/ITO/glass surface.

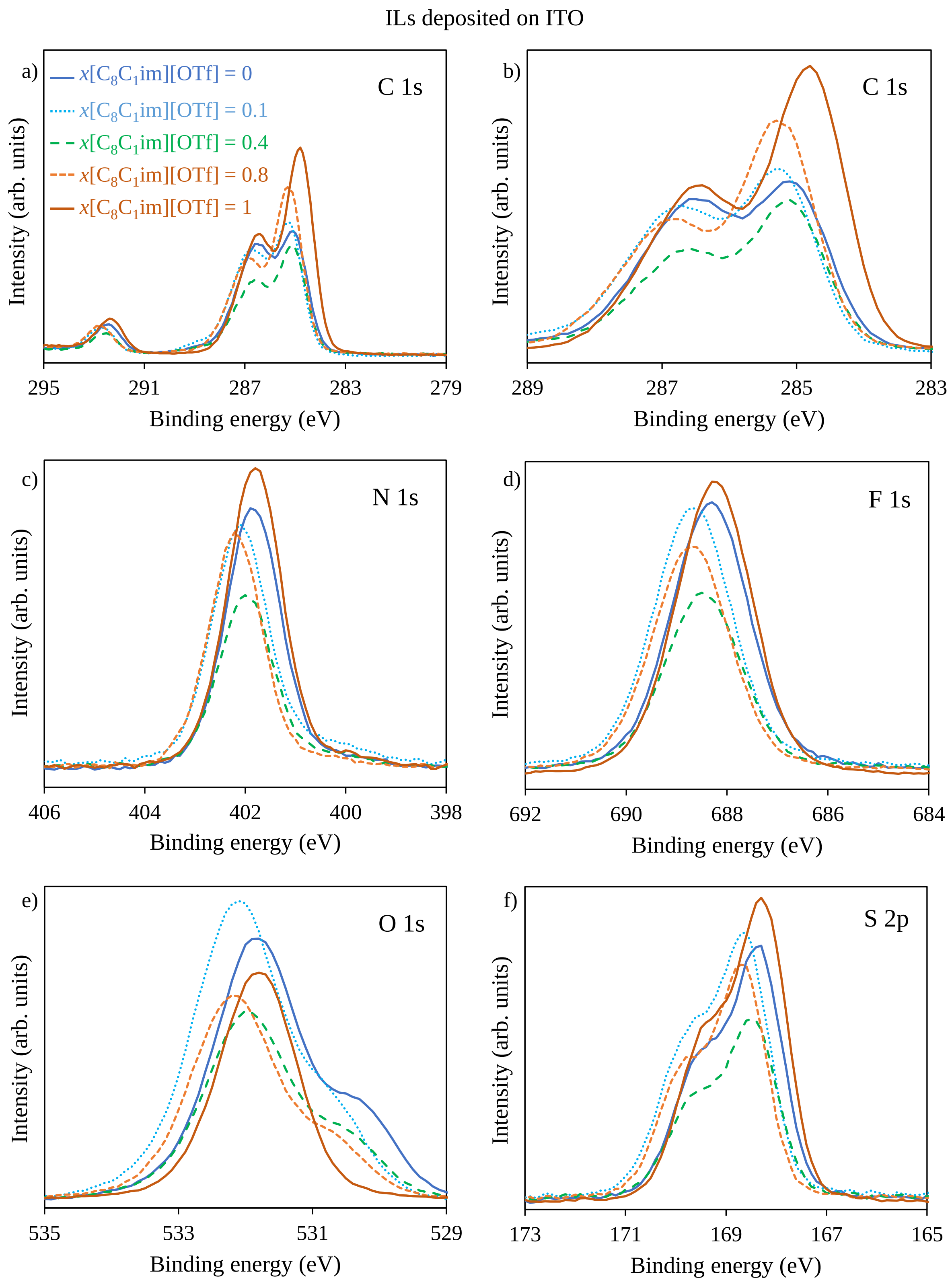

Figure S29. High-resolution XPS spectra of IL films deposited on ITO/glass surfaces by simultaneous deposition of [C_2C_1im][OTf] and [C_8C_1im][OTf], with a varying mole fraction (*x*) of each IL in the resulting film: *x*{[C_8C_1im][OTf]} = 0 (blue solid curve); *x*{[C_8C_1im][OTf]} = 0.1 (blue dashed curve); *x*{[C_8C_1im][OTf]} = 0.4 (green dashed curve); *x*{[C_8C_1im][OTf]} = 0.8 (dashed brown curve); *x*{[C_8C_1im][OTf]} = 1 (solid brown curve). The XPS spectra were acquired for C 1s (a and b), N 1s (c), F 1s (d), O 1s (e), and S 2p (f).

ILs deposited on Au

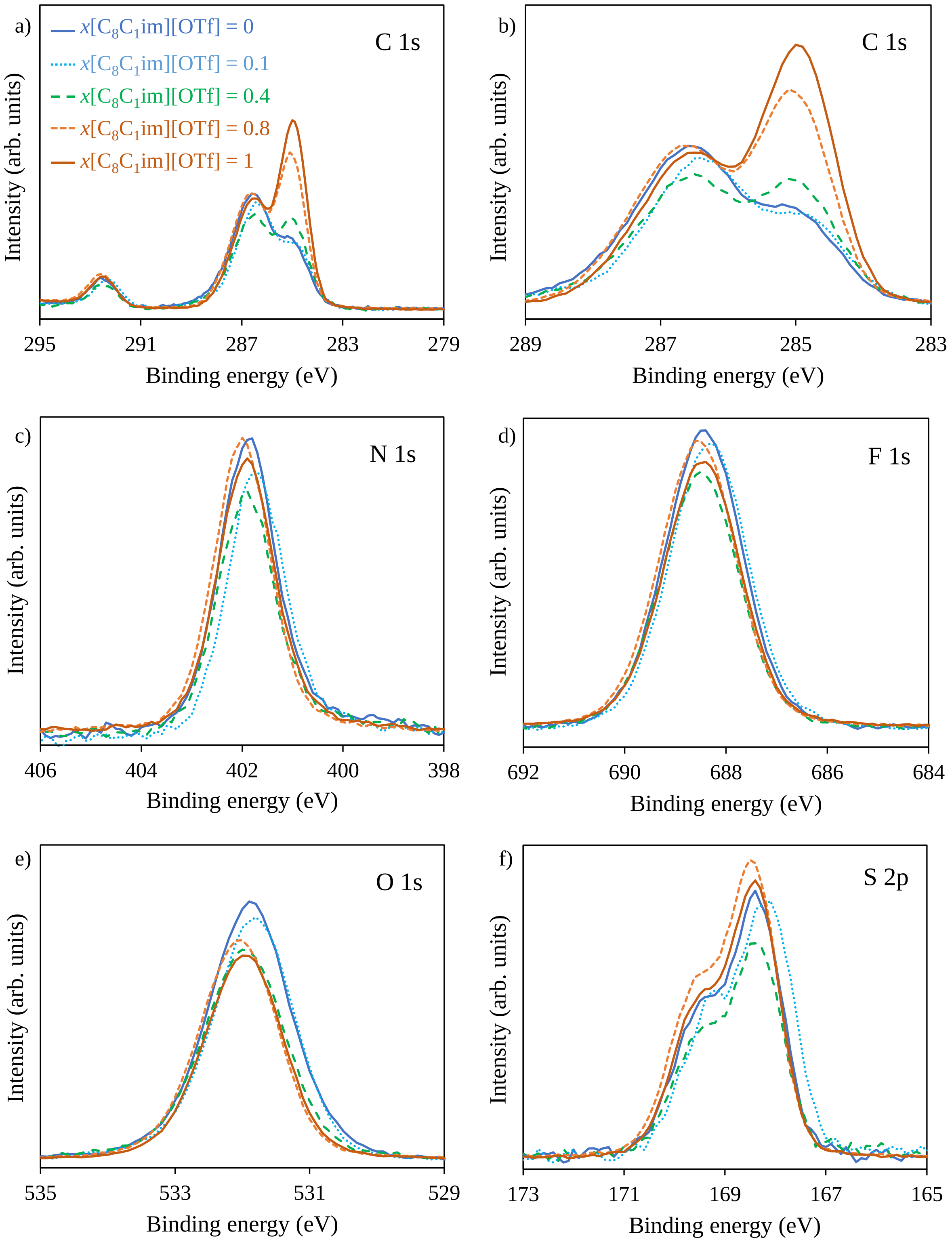

Figure S30. High-resolution XPS spectra of IL films deposited on Au/ITO/glass surfaces by simultaneous deposition of [C_2C_1im][OTf] and [C_8C_1im][OTf], with a varying mole fraction (x) of each IL in the resulting film: x{[C_8C_1im][OTf]} = 0 (blue solid curve); x{[C_8C_1im][OTf]} = 0.1 (blue dashed curve); x{[C_8C_1im][OTf]} = 0.4 (green dashed curve); x{[C_8C_1im][OTf]} = 0.8 (dashed brown curve); x{[C_8C_1im][OTf]} = 1 (solid brown curve). The XPS spectra were acquired for C 1s (a and b), N 1s (c), F 1s (d), O 1s (e), and S 2p (f).

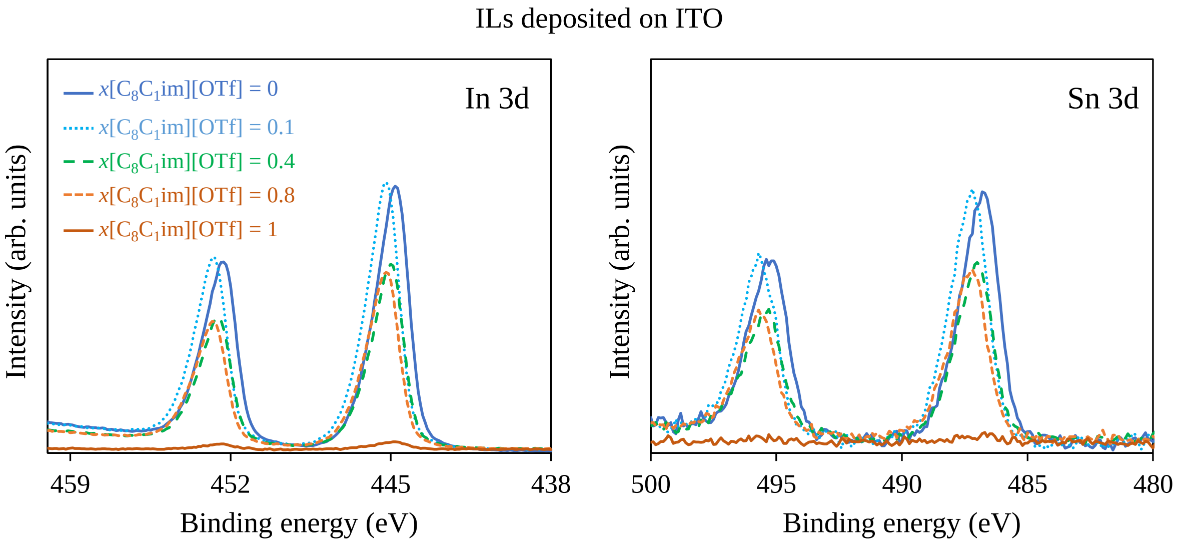

Figure S31. High-resolution XPS spectra of IL films deposited on ITO/glass surfaces by simultaneous deposition of [C_2C_1im][OTf] and [C_8C_1im][OTf], with a varying mole fraction (*x*) of each IL in the resulting film: *x*{[C_8C_1im][OTf]} = 0 (blue solid curve); *x*{[C_8C_1im][OTf]} = 0.1 (blue dashed curve); *x*{[C_8C_1im][OTf]} = 0.4 (green dashed curve); *x*{[C_8C_1im][OTf]} = 0.8 (dashed brown curve); *x*{[C_8C_1im][OTf]} = 1 (solid brown curve). The XPS spectra were acquired for In 3d and Sn 3d.

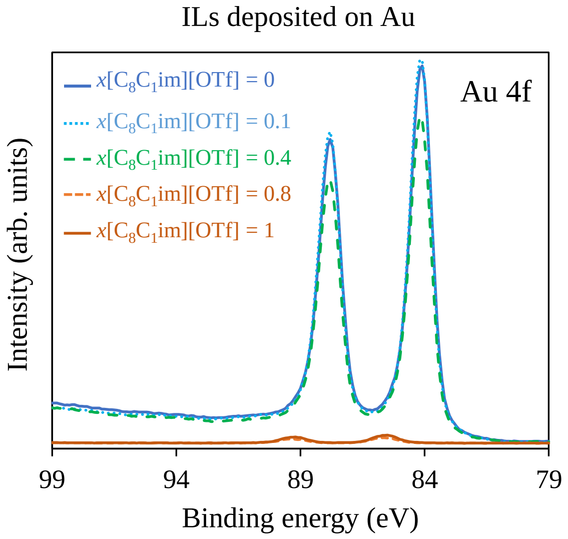

Figure S32. High-resolution XPS spectra of IL films deposited on Au/ITO/glass surfaces by simultaneous deposition of [C_2C_1im][OTf] and [C_8C_1im][OTf], with a varying mole fraction (*x*) of each IL in the resulting film: *x*{[C_8C_1im][OTf]} = 0 (blue solid curve); *x*{[C_8C_1im][OTf]} = 0.1 (blue dashed curve); *x*{[C_8C_1im][OTf]} = 0.4 (green dashed curve); *x*{[C_8C_1im][OTf]} = 0.8 (dashed brown curve); *x*{[C_8C_1im][OTf]} = 1 (solid brown curve). The XPS spectra were acquired for Au 4f.

Table S2. Experimental {C_{cation} : C_{anion}}, {C_{cation} : N_{cation}}, {N_{cation} : F_{anion}}, and {N_{cation} : S_{anion}} ratios derived from the XPS data. For pure ILs ([C_2C_1im][OTf]:[C_8C_1im][OTf] (1:0) and [C_2C_1im][OTf]:[C_8C_1im][OTf] (0:1)) the expected/empiric values based on a cation-to-anion ratio of 1:1 are presented for comparison.

IL mixture	C_{cation} : C_{anion}	C_{cation} : N_{cation}	N_{cation} : F_{anion}	N_{cation} : S_{anion}
substrate: ITO/glass				
**[C_2C_1im][OTf]:[C_8C_1im][OTf] (1:0)**	10.0 : 1 [a)]<br>6 : 1 (empiric)	10.0 : 2 [a)]<br>6 : 2 (empiric)	1.6 : 3<br>2 : 3 (empiric)	1.9 : 1<br>2 : 1 (empiric)
**[C_2C_1im][OTf]:[C_8C_1im][OTf] (0.9:0.1)**	12.1 : 1	11.2 : 2	1.6 : 3	1.7 : 1
**[C_2C_1im][OTf]:[C_8C_1im][OTf] (0.6:0.4)**	12.6 : 1	11.7 : 2	1.7 : 3	1.8 : 1
**[C_2C_1im][OTf]:[C_8C_1im][OTf] (0.2:0.8)**	13.3 : 1	13.1 : 2	1.7 : 3	1.8 : 1
**[C_2C_1im][OTf]:[C_8C_1im][OTf] (0:1)**	12.2 : 1<br>12.0 : 1 (empiric)	12.0 : 2<br>12 : 2 (empiric)	1.7 : 3<br>2 : 3 (empiric)	1.8 : 1<br>2 : 1 (empiric)
substrate: Au/ITO/glass				
**[C_2C_1im][OTf]:[C_8C_1im][OTf] (1:0)**	8.2 : 1 [a)]<br>6.0 : 1 (empiric)	7.9 : 2 [a)]<br>6 : 2 (empiric)	1.6 : 3<br>2 : 3 (empiric)	2.1 : 1<br>2 : 1 (empiric)
**[C_2C_1im][OTf]:[C_8C_1im][OTf] (0.9:0.1)**	7.9 : 1	7.8 : 2	1.6 : 3	1.9 : 1
**[C_2C_1im][OTf]:[C_8C_1im][OTf] (0.6:0.4)**	9.7 : 1	9.3 : 2	1.6 : 3	2.1 : 1
**[C_2C_1im][OTf]:[C_8C_1im][OTf] (0.2:0.8)**	10.8 : 1	10.7 : 2	1.7 : 3	1.8 : 1
**[C_2C_1im][OTf]:[C_8C_1im][OTf] (0:1)**	12.4 : 1<br>12 : 1 (empiric)	12.0 : 2<br>12 : 2 (empiric)	1.7 : 3<br>2 : 3 (empiric)	1.9 : 1<br>2 : 1 (empiric)

[a)] These values differ from the empirical ratio as they also take into account the substrate information, especially the presence of adventitious carbon contamination.

6. UV-Vis Spectroscopy

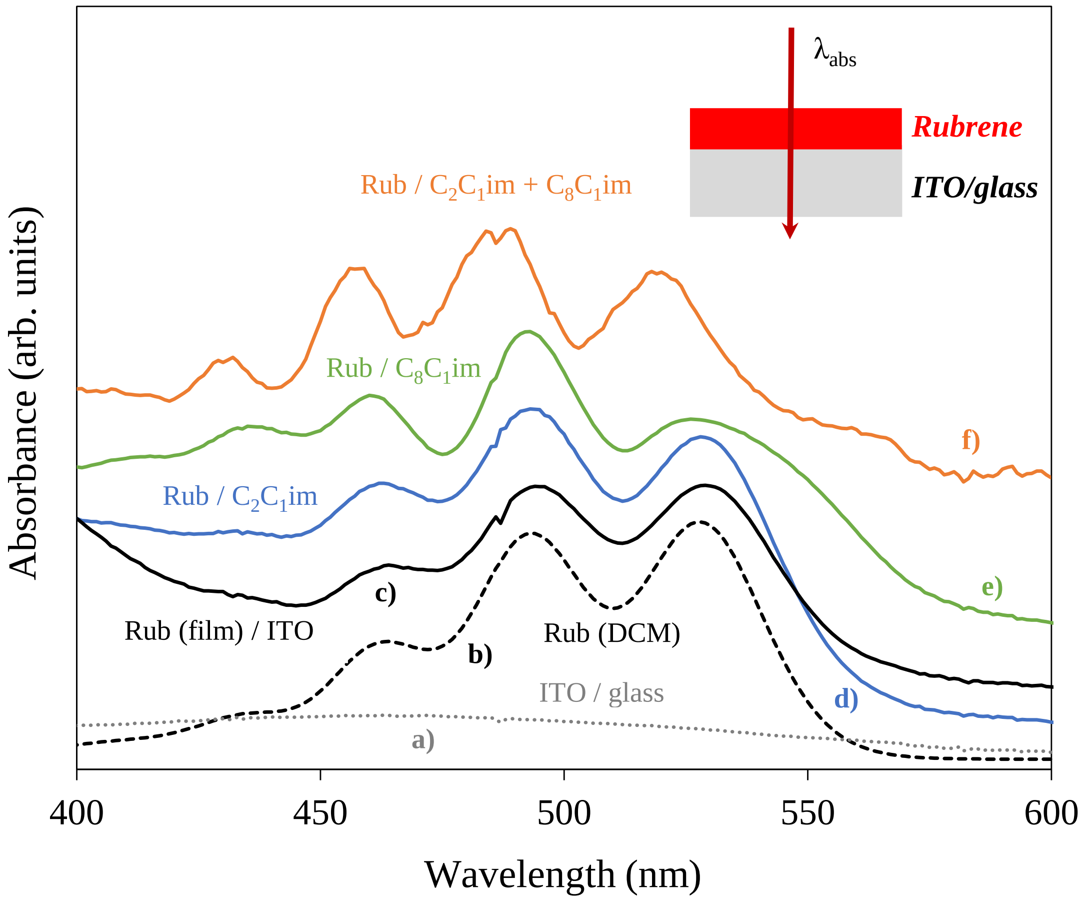

Figure S33. UV-vis absorption spectra comparison of rubrene (Rub) films (200 nm thick) deposited on various surfaces: Rub deposited on ITO/glass (c); Rub deposited on ITO/glass pre-coated with $[C_2C_1im][OTf]$ (d); Rub deposited on ITO/glass pre-coated with $[C_8C_1im][OTf]$ (e); Rub deposited on ITO/glass pre-coated with a mixture of $[C_2C_1im][OTf]$ (x=0.6) and $[C_8C_1im][OTf]$ (x=0.4) (f). For comparison, the UV-vis spectrum of the ITO/glass substrate (a) and the UV-vis spectrum of Rub solution ([Rub] ≈ 10^{-5} mol·dm^{-3}) in DCM (b) are also included.

7. X-Ray Diffraction

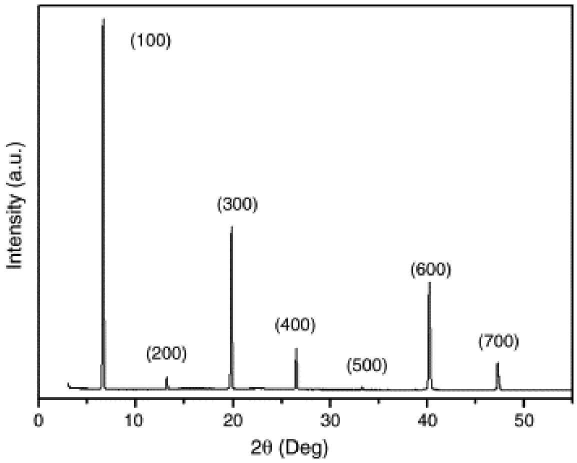

Figure S34. Literature data of the XRD pattern of rubrene single crystals.[11]

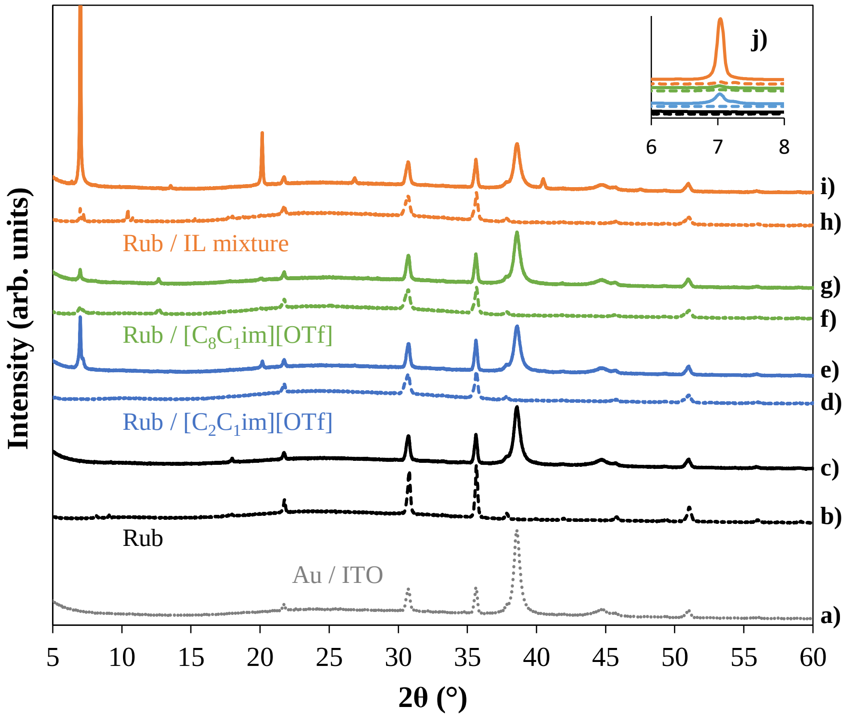

Figure S35. X-ray diffraction (XRD) patterns of rubrene films deposited on various surfaces: (a) XRD pattern of the Au/ITO substrate; (b) XRD pattern of rubrene (Rub) film deposited on ITO/glass; (c) XRD pattern of rubrene film deposited on Au/ITO/glass; (d) XRD pattern of rubrene film deposited on ITO previously coated with [C_2C_1im][OTf]; (e) XRD pattern of rubrene film deposited on Au/ITO previously coated with [C_2C_1im][OTf]; (f) XRD pattern of rubrene film deposited on ITO previously coated with [C_8C_1im][OTf]; (g) XRD pattern of rubrene film deposited on Au/ITO previously coated with [C_8C_1im][OTf]; (h) XRD pattern of rubrene film deposited on ITO previously coated with a mixture of [C_2C_1im][OTf] (x=0.6) and [C_8C_1im][OTf] (x=0.4); (i) XRD pattern of rubrene film deposited on Au/ITO previously coated with a mixture of [C_2C_1im][OTf] (x=0.6) and [C_8C_1im][OTf] (x=0.4); (j) Zoomed-in XRD pattern of the peak (100) in the 2θ range of 6-8°.

Estimation of crystallite sizes:[12,13]

The crystallite sizes were determined using two methods: the traditional approach, which applies the Scherrer equation to individual peaks and then averages the results, and the Williamson-Hall (W-H) representation. In both cases, approximately six peaks were considered. The Scherrer equation typically provides a smaller crystallite size *D* than the Williamson-Hall plot when residual stress is present. This is because stress increases peak width; thus, relying solely on the Scherrer equation means using an excessively large full width at half-maximum (*FWHM*), which results in an underestimated crystallite size. The Scherrer equation used is:

$$D = \frac{k \cdot \lambda}{\beta \cdot \cos\theta}$$

where *k* is the Debye–Scherrer constant (~0.94 for spherical crystallites), λ is the incident X-ray wavelength, θ is the diffraction angle, and β is the *FWHM* of the XRD peak. With the constants substituted, the equation simplifies to:

$$D = \frac{0.94 \cdot 0.15406}{\mathrm{radians}(FWHM) \cdot \cos(\mathrm{radians}(2\theta/2))}$$

for each *FWHM*-2θ peak pair. One of the weaknesses of the Scherrer method is that it only correlates peak width with crystallite size. However, peak width depends on crystallite size and lattice strain, which arises due to various structural defects such as point defects, grain boundaries, and stacking faults. The W-H method accounts for these factors, providing a more comprehensive estimation of crystallite size. The average crystallite size of the particles (d_{XRD}) using the W-H method was calculated as:

$$\beta_{\mathrm{total}} = \beta_{\mathrm{size}} + \beta_{\mathrm{strain}} = \frac{k \cdot \lambda}{d_{\mathrm{XRD}} \cdot \cos(\theta)} + 4 \cdot \eta \cdot \tan(\theta)$$

where β_{total} is the *FWHM* of the XRD peak (including both instrumental and sample-dependent effects), *k* is the Debye–Scherrer constant (~0.94 for spherical crystallites), λ is the incident X-ray wavelength, θ is the diffraction angle, and η is the microstrain parameter. For all samples, the microstrain was considered negligible (η<0.1 %), meaning it does not significantly impact the primary physical properties of the bulk sample. A plot of $\frac{\beta_{\mathrm{total}} \cdot \cos(\theta)}{k \cdot \lambda}$ vs. $\frac{4 \cdot \mathrm{sen}(\theta)}{k \cdot \lambda}$ was used to approximate the average crystallite size, which was extracted from the inverse of the y-intercept.

8. Experimental Data for the Deposition of ILs

Table S3. Experimental data for the deposition of IL mixtures with varying proportions of each IL and different film thicknesses. Parameters include: effusion temperature ($T_{eff.}$); substrate temperature ($T_{subst.}$); deposition rate of each individual IL (φ) and the total deposition rate from the simultaneous deposition of both ILs (φ (total)); deposition time; and film thickness.

IL mixture	$T_{eff.}$ [a]	$T_{subst.}$ [b]	φ	φ (total)	Deposition time	Film Thickness
	K	K	Å·s^{-1}	Å·s^{-1}	min	ML
**[C_2C_1im][OTf]:[C_8C_1im][OTf] (1:0)**						
[C_2C_1im][OTf] (x = 1)	528 ± 3	283.2	0.6	0.6 ± 0.1	18.9	100
[C_8C_1im][OTf] (x = 0)	N.A.		0			
[C_2C_1im][OTf] (x = 1)	528 ± 3		0.6	0.6 ± 0.1	9.4	50
[C_8C_1im][OTf] (x = 0)	N.A.		0			
**[C_2C_1im][OTf]:[C_8C_1im][OTf] (0.9:0.1)**						
[C_2C_1im][OTf] (x = 0.89)	523 ± 3	283.2	0.5	0.6 ± 0.1	19.2	100
[C_8C_1im][OTf] (x = 0.11)	493 ± 3		0.1			
[C_2C_1im][OTf] (x = 0.89)	523 ± 3		0.5	0.6 ± 0.1	9.6	50
[C_8C_1im][OTf] (x = 0.11)	493 ± 3		0.1			
**[C_2C_1im][OTf]:[C_8C_1im][OTf] (0.8:0.2)**						
[C_2C_1im][OTf] (x = 0.76)	520 ± 3	283.2	0.4	0.6 ± 0.1	19.6	100
[C_8C_1im][OTf] (x = 0.24)	506 ± 3		0.2			
[C_2C_1im][OTf] (x = 0.76)	520 ± 3		0.4	0.6 ± 0.1	9.8	50
[C_8C_1im][OTf] (x = 0.24)	506 ± 3		0.2			
**[C_2C_1im][OTf]:[C_8C_1im][OTf] (0.6:0.4)**						
[C_2C_1im][OTf] (x = 0.61)	516 ± 3	283.2	0.3	0.6 ± 0.1	20.0	100
[C_8C_1im][OTf] (x = 0.39)	514 ± 3		0.3			
[C_2C_1im][OTf] (x = 0.61)	516 ± 3		0.3	0.6 ± 0.1	10.0	50
[C_8C_1im][OTf] (x = 0.39)	514 ± 3		0.3			
**[C_2C_1im][OTf]:[C_8C_1im][OTf] (0.4:0.6)**						
[C_2C_1im][OTf] (x = 0.44)	510 ± 3	283.2	0.2	0.6 ± 0.1	20.4	100
[C_8C_1im][OTf] (x = 0.56)	518 ± 3		0.4			
[C_2C_1im][OTf] (x = 0.44)	510 ± 3		0.2	0.6 ± 0.1	10.2	50
[C_8C_1im][OTf] (x = 0.56)	518 ± 3		0.4			
**[C_2C_1im][OTf]:[C_8C_1im][OTf] (0.2:0.8)**						
[C_2C_1im][OTf] (x = 0.24)	500 ± 3	283.2	0.1	0.6 ± 0.1	21.0	100
[C_8C_1im][OTf] (x = 0.76)	522 ± 3		0.5			
[C_2C_1im][OTf] (x = 0.24)	500 ± 3		0.1	0.6 ± 0.1	10.5	50
[C_8C_1im][OTf] (x = 0.76)	522 ± 3		0.5			
**[C_2C_1im][OTf]:[C_8C_1im][OTf] (0:1)**						
[C_2C_1im][OTf] (x = 0)	N.A.	283.2	0	0.6 ± 0.1	21.7	100
[C_8C_1im][OTf] (x = 1)	528 ± 3		0.6			
[C_2C_1im][OTf] (x = 0)	N.A.		0	0.6 ± 0.1	10.8	50
[C_8C_1im][OTf] (x = 1)	528 ± 3		0.6			

[a] The vapor pressures of [C_2C_1im][OTf] and [C_8C_1im][OTf] at the studied evaporation temperatures are estimated to be within the interval between 0.01 and 1 Pa. The ILs were evaporated from Knudsen effusion cells through an orifice with a diameter of 3 mm.

[b] The depositions were performed simultaneously on ITO, Ag/ITO, and Au/ITO substrates.

References

(1) Klomfar, J.; Součková, M.; Pátek, J. Temperature Dependence Measurements of the Density at 0.1 MPa for 1-Alkyl-3-Methylimidazolium-Based Ionic Liquids with the Trifluoromethanesulfonate and Tetrafluoroborate Anion. *J. Chem. Eng. Data* **2010**, *55*, 4054–4057.

(2) Nebig, S.; Gmehling, J. Measurements of Different Thermodynamic Properties of Systems Containing Ionic Liquids and Correlation of These Properties Using Modified UNIFAC (Dortmund). *Fluid Ph. Equilib.* **2010**, *294*, 206–212.

(3) Anwar, N.; Riyazuddeen. Excess Molar Volumes, Excess Molar Isentropic Compressibilities, Viscosity Deviations, and Activation Parameters for 1-Ethyl-3-MethylImidazolium Trifluoro-Methanesulfonate + Dimethyl Sulfoxide And/or Acetonitrile at T = 298.15 to 323.15 K and p = 0.1 MPa. *J. Chem. Eng. Data* **2018**, *63*, 269–289.

(4) Diejomaoh Abafe, O. T.; Azim, M. M.; Martincigh, B. S.; Stark, A. Cation-Fluorinated Ionic Liquids: Synthesis, Physicochemical Properties and Comparison with Non-Fluorinated Analogues. *J. Mol. Liq.* **2022**, *349*, 118104.

(5) Every, H.; Bishop, A. G.; Forsyth, M.; MacFarlane, D. R. Ion Diffusion in Molten Salt Mixtures. *Electrochim. Acta* **2000**, *45*, 1279–1284.

(6) Information obtained from IoLiTec GmbH. Available at https://iolitec.de/en/node/141. Accessed on October 16, 2024.

(7) Almeida, H. F. D.; Teles, A. R. R.; Lopes-da-Silva, J. A.; Freire, M. F.; Coutinho, J. A. P. Influence of the Anion on the Surface Tension of 1-Ethyl-3-methylimidazolium-Based Ionic Liquids. *J. Chem. Thermodyn.* **2012**, *54*, 49−54.

(8) Tariq, M.; Freire, M. G.; Saramago, B.; Coutinho, J. A. P.; Lopes, J. N. C.; Rebelo, L. P. N. Surface Tension of Ionic Liquids and Ionic Liquid Solutions. *Chem. Soc. Rev.* **2012**, *41*, 829–868.

(9) Costa, J. C. S.; Rocha, R. M.; Vaz, I. C. M.; Torres, M. C.; Mendes, A.; Santos, L. M. N. B. F. Description and Test of a New Multilayer Thin Film Vapor Deposition Apparatus for Organic Semiconductor Materials. *J. Chem. Eng. Data* **2015**, *60*, 3776–3791.

(10) Costa, J. C. S.; Coelho, A. F. S. M. G.; Mendes, A.; Santos, L. M. N. B. F. Nucleation and Growth of Microdroplets of Ionic Liquids Deposited by Physical Vapor Method onto Different Surfaces. *Appl. Surf. Sci.* **2018**, *428*, 242–249.

(11) Zeng, X.; Zhang, D.; Duan, L.; Wang, L.; Dong, G.; Qiu, Y. Morphology and Fluorescence Spectra of Rubrene Single Crystals Grown by Physical Vapor Transport. *Appl. Surf. Sci.* **2007**, *253*, 6047–6051.

(12) Cullity, B. D.; Smoluchowski, R. Elements of X-ray Diffraction. *Phys. Today* **1957**, *10*, 50.

(13) Hassanzadeh-Tabrizi, S. A. Precise calculation of crystallite size of nanomaterials: A review. *J. Alloys Compd.* **2023**, *968*, 171914.
